# Bottom-Up Engineering Strategies for High-Performance Thermoelectric Materials

**DOI:** 10.1007/s40820-021-00637-z

**Published:** 2021-05-03

**Authors:** Qiang Zhu, Suxi Wang, Xizu Wang, Ady Suwardi, Ming Hui Chua, Xiang Yun Debbie Soo, Jianwei Xu

**Affiliations:** 1 Institute of Materials Research and Engineering, A*STAR (Agency for Science, Technology and Research), 2 Fusionopolis Way, Innovis, #08-03, Singapore, 138634 Singapore; 2 Department of Chemistry, National University of Singapore, 3 Science Drive 3, Singapore, 117543 Singapore

**Keywords:** Thermoelectric, Nanostructures, Bottom-up, Synthesis, Nanomaterials

## Abstract

The recent advancements in thermoelectric materials are largely credited to two factors, namely established physical theories and advanced materials engineering methods. The developments in the physical theories have come a long way from the “phonon glass electron crystal” paradigm to the more recent band convergence and nanostructuring, which consequently results in drastic improvement in the thermoelectric figure of merit value. On the other hand, the progresses in materials fabrication methods and processing technologies have enabled the discovery of new physical mechanisms, hence further facilitating the emergence of high-performance thermoelectric materials. In recent years, many comprehensive review articles are focused on various aspects of thermoelectrics ranging from thermoelectric materials, physical mechanisms and materials process techniques in particular with emphasis on solid state reactions. While bottom-up approaches to obtain thermoelectric materials have widely been employed in thermoelectrics, comprehensive reviews on summarizing such methods are still rare. In this review, we will outline a variety of bottom-up strategies for preparing high-performance thermoelectric materials. In addition, state-of-art, challenges and future opportunities in this domain will be commented.

## Highlights

- Recent advances of various bottom-up approaches for constructing nanostructured semiconductor thermoelectric materials with different dimensions are reviewed.
- The relationships between the nanostructures and the key electronic and thermal transport parameters contributing to ZT are discussed.
- The challenges of the bottom-up strategies and suggestions for future development toward thermoelectric applications are provided.

## Graphical abstract

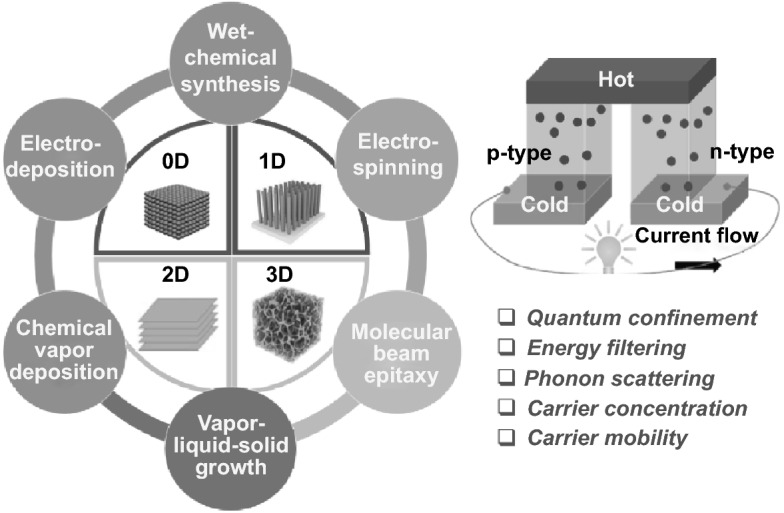

## Introduction

The ever-increasing demand on electricity has driven the expansion of electricity supply sources, such as solar energy, nuclear power and photovoltaics. All these electricity sources are alternatives to the conventional fossil fuels. However, all these power generation approaches cannot address more than 60% of energy loss worldwide as waste heat. The ubiquity of low-grade waste heat (< 200 °C) in modern electronic devices is an opportunity in terms of energy recovery. In a bid to utilize the waste heat, thermoelectrics becomes is a viable option as it converts a thermal gradient into electrical energy in the solid state. This type of conversion from thermal heat to electricity possesses a lot of advantages, such as silent mode, no noise and less pollution, which has also triggered the development of various niche applications, including radioisotope thermoelectric (TE) generators (TEGs) for the space exploration, for example, National Aeronautics and Space Administration’s Voyager 1 and 2. However, all these applications put forward strict requirements for energy conversion efficiency, and traditionally, high-performance inorganic semiconducting materials, such as bulk Bi_2_Te_3,_ SnSe, and PbTe [1–6], have been extensively studied and fabricated into the TEGs.

The efficiency of TE materials can be expressed by dimensionless figure of merit (*ZT* = *σS*^2^*T*/*κ*), where *S, σ,* *T*, and *κ* represent Seebeck coefficient, electrical conductivity, absolute temperature, and thermal conductivity, respectively. In order to enhance *ZT*, a large *S*, a high *σ,* and a lower *κ* are preferred, but these parameters are inter-dependent on each other. For instance, while the *S* has an inverse dependency on the carrier concentration (*n*), the *σ* is proportional to the *n.* To optimize the power factor (*PF* = *σS*^*2*^) and *ZT*, the *n* needs to be optimized either via doping or defect engineering to be around 10^19^ cm^−3^ level [7, 8]. However, the introduced dopants can deteriorate the charge carrier mobility, thus hindering the magnitude of the optimal power factor [9, 10]. A popular and effective strategy for enhancing the *PF* is via the electronic band-engineering [11–16]. In the electronic band-engineering, either the band-convergence or the effective mass manipulation can be explored to increase the *S* or *σ*, respectively [13, 17, 18]. In addition to the *PF*, the *κ* is also an important parameter that affects the *ZT* value. The *κ* is composed of electrical thermal conductivity (*κ*_e_) and lattice thermal conductivity (*κ*_L_). The *κ*_e_ value is predominantly affected by the *σ* through the charge carrier concentration and the *κ*_L_ is controlled by the introduced impurities through various phonon scattering mechanisms. The close inter-correlation between the *S*, *σ,* and *κ* makes it highly challenging to achieve a high *ZT* value.

Another useful approach to improve *ZT* is to prepare nanostructured semiconducting materials. The advantages of nanostructuring TE materials offer a pathway to positively decouple the correlation between the *S*, *σ,* and *κ*. The quantum confinement effects associating with these nanostructures help to alter the electronic density-of-states, and therefore improve the *PF* [19]. In addition, another noteworthy feature of nanostructuring is that it is able to decrease the *κ*_L_ through respective phonon scattering effects. For instance, one approach to reduce the *κ*_L_ is to minimize the phonon relaxation time through the introduction of phonon scattering sources such as point defects, dislocations and interfaces [20].

**Fig. 1:**
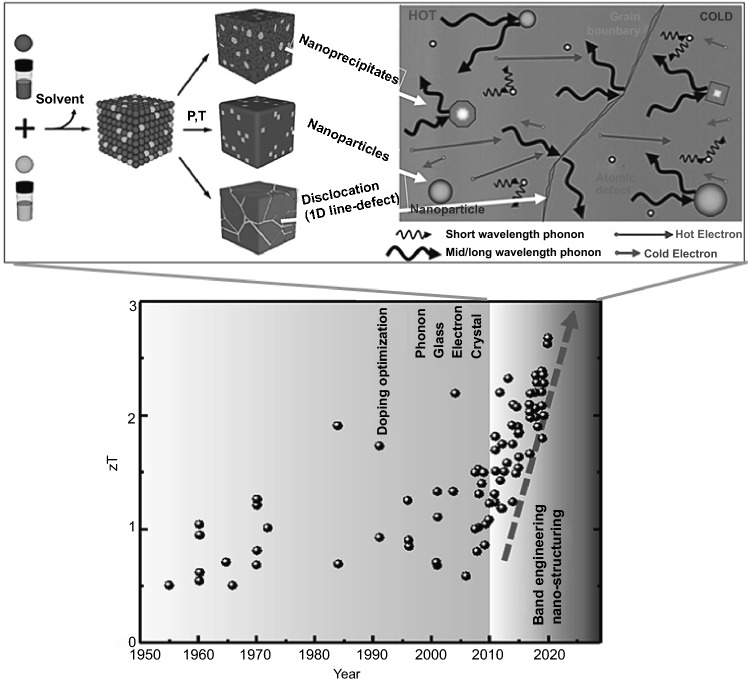
Illustrations showing the bottom-up assembly to synthesize nanoscaled thermoelectric materials and the advantageous effects brought about by multi-wavelength phonon scattering due to the various shape, and size defects brought about by these bottom-up approaches. Adapted with permission from Ref. [22, 27]

Traditionally, a popular method to prepare nanostructured TE materials is to make use of a top-down approach, including ball milling, exfoliation, annealing, and so on. However, most of these processes are energy-consuming. Additionally, the precise control of the particle size, size-distribution and shape remains challenging. In order to address these issues, bottom-up approaches is sought after. The primary advantage of bottom-up approach lies in its versatility in designing nanostructured materials, which is favorable for phonon scattering (i.e., lower thermal conductivity). Approaches such as liquid-phase synthesis, vapor–liquid–solid (VLS) growth, solution–liquid–solid (SLS) growth, chemical vapor deposition (CVD), electrochemical deposition, electrospinning, and so on have provided a wide variety of nanoscaled architectures, offering many advantages over some traditional methods in controlling phase purity, crystallinity, density and dimensions of nanostructures. For nanomaterial-based thermoelectric devices, the nanoscaled particles can be assembled at a macroscopic scale and homogeneously distributed, either in thin film or in bulk materials. The final thermoelectric properties will very much depend on not only the individual nanoparticles, but also the manner they assemble. For instance, by controlling annealing temperature and/or pressure, various nano-sized defects can be engineered, as shown in Fig. 1. Consequently, by engineering multiscale defects such as point defect (nanoparticle), nano-precipitates, and line dislocations, it is possible to scatter phonons of a wide-range of wavelengths, resulting in very low *κ*_L_. Another advantage of bottom-up approaches is that they generally do not require a substrate to support the growth, therefore enabling higher throughput than traditional methods such as physical vapor deposition. This paper will cover the recent studies of using various nanostructures to tune the TE performance through bottom-up engineering strategies, which have, however, not extensively been attracted attention in existing reviews on the nanostructured TE materials [8, 21–26].

## Benefits of Nanostructuring

Understanding the inter-correlation between different TE parameters that make up the *ZT* can be a useful guide to design nanostructured TE materials. Through extensive studies on the electron and phonon transport and electron–phonon couplings, multiple strategies have been investigated and applied for the enhancement of the TE performance. In this section, these strategies including size effect, mean free path and quantum effect, energy filtering effect at grain boundary, phonon scattering effect by nanoparticles as well as other related effects, will be summarized in details, embodying the latest understanding and effective manipulation of the interplay among carrier, phonon, lattice, interface and electronic states in TE nanomaterials.

### Nanostructuring Effect in Thermoelectrics

Conceptually, in order to obtain a high *ZT* value, both the *S* and *σ* must be large, while the *κ* must be minimized so as to maximize the power output at a high temperature difference. Traditionally, there are two main design principles in searching for bulk TE materials with high *ZT* values. The first approach is the “phonon glass electron crystal” approach, and the other approach is the nanostructuring of TE materials. To achieve the phonon glass electron crystal, materials with complex structures are in general preferred [11, 28–31]. Recently, TE materials with exceptionally high *ZT* values of above 2 have been reported owing to the advance of various materials development approaches, including band engineering, defect optimization and nanostructuring. Nanostructuring is a promising way to improve the TE performance by means of reduction of the characteristic length of the phonons mean-free paths [8, 32–34]. It is generally accepted that the mean-free paths for phonons are much longer than that of electrons, and therefore by judiciously tuning the nanostructure size to the same order as the phonon mean-free paths, it is possible to selectively scatter phonons and not electrons, resulting in a lower *κ*_L_ while maintaining a high *σ* [35, 36].

The classical nanostructuring effects concern the scattering-limited mean free paths and the confinement-induced variation in the electronic dispersion relation, respectively. For instance, heat is carried by phonons with a wide, momentum and energy temperature-dependent spectrum, prohibiting the *k*_L_ by limiting the phonon mean-free path over a broad temperature range therefore requiring all-scale hierarchical nanostructuring and microstructuring [37]. Under the circumstance of the classical size effect, the lowest possible *k*_L_ is defined as the amorphous limit, when the phonon mean-free path gets as small as the interatomic spacing and heat is carried by the random-walking Einstein mode, just like in amorphous materials. The minimum lattice thermal conductivity *k*_*min*_ can be expressed as [38]:

1 $$\kappa _{{\min }} = (\frac{\pi }{6})^{{\frac{1}{3}}} k_{B} n_{a}^{{\frac{2}{3}}} \mathop \sum \limits_{i} \nu _{i} \left( {\frac{T}{{\theta _{i} }}} \right)^{2} \mathop \smallint \limits_{0}^{{\theta _{i} /T}} \frac{{x^{3} e^{x} }}{{\left( {e^{x} - 1} \right)^{2} }}$$

where the ${\theta }_{i}={\nu }_{i}(\hbar /{\mathsf{k}}_{B}){(6{\pi }^{2}{n}_{a})}^{1/3}$ is the cutoff frequency, ${n}_{a}$ is the number of atoms per unit volume, ħ is the Planck’s constant *h* divided by 2π, and ${\upnu }_{i}$ is the sound velocity vector.

Theoretical and experimental reports show that the high density of nanoscaled grains can induce the effective phonon scattering of over 60% and remarkably reduce the *κ* [39]. In addition, due to the low dimension of nanostructures, the phonon confinement effects must be considered. For instance, the Umklapp scattering process in superlattice is different from those of bulk materials. The so-called mini-Umklapp arises due to the periodically alternating layers of a material that has a large superlattice constant, resulting in a mini-Brillouin zone, and hence a lower *κ* [40].

### Quantum Effect

**Fig. 2:**
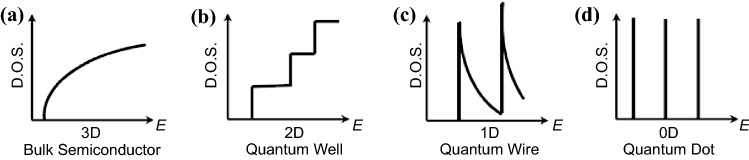
Illustrations showing the electronic density-of-states of bulk and low dimensional materials. **a** Bulk semiconductor. **b** 2D materials. **c** 1-D materials (i.e., nanowires (NWs), nanorods). **d** Quantum dots. Adapted with permission from Ref. [47]

Low dimensional materials/nanostructures are defined by a characteristic dimension in order comparable to the de Broglie wavelength of charge carriers. As a result, the degree of freedom of the carrier motion is restricted by the dimension of the nanostructures. Consequently, the electronic transport behavior is drastically altered, resulting in the so-called quantum size effect [41]. Figure 2 illustrates the electronic band structure behavior of various low dimensional materials as compared with traditional three-dimensional (3D) bulk materials. Compared with 3D materials, all low dimensional materials have a very sharp feature in the density of states, especially for one-dimensional (1D) and zero-dimensional (0D) materials. These features are very beneficial to enhance the *S* and thus the *PF* [42, 43]. Therefore, this quantum size effect underlies the paradigm of TE nanostructuring [44–46].

To understand the origin of the *S* enhancement in low dimensional materials, it is helpful to look into the Mott equation shown below:

2 $$S= \frac{{\pi }^{2}{\kappa }_{B}^{2}T}{3e}{\left[\frac{1}{n}\frac{\partial n(E)}{\partial E}+\frac{1}{\mu }\frac{\partial \mu (E)}{\partial E}\right]}_{E={E}_{F}}$$

The first and second terms inside the square bracket represent the contribution to the *S* due to the carrier concentration modulation and carrier mobility modulation, respectively. In most TE studies, the second term (carrier mobility modulation) is ignored because of the low energy dependence of mobility (i.e., *r* = − 0.5 for the acoustic phonon scattering). The density of states *vs* the energy profile shown in Fig. 2 is directly proportional to the first term in Eq. 2, $\partial n(E)/\partial E$. Therefore, a higher slope in the density of states *vs* the energy will result in a higher *S*. Theoretically, the enhancement in the *S* in low dimensional materials has been predicted in several literatures for 2D superlattice as well as 1D NWs [48–52]. More recently, numerous experimental enhancements on the *S* in low dimensional materials have been reported, such as Pb_1−*x*_Eu_*x*_Te/PbTe multiple quantum wells and PbTe_1−*x*_Se_*x*_/PbTe quantum dot superlattice [53, 54].

In addition to low dimensional materials, the concept of enhancing the slope of density of states *vs* the energy has been successfully applied using resonant doping in bulk 3D materials. Most notably, elements Tl and In have been shown to be a resonant dopant for PbTe and GeTe, respectively [12, 55, 56]. These observation suggests that physical intuition derived from the studies of low dimensional materials can also be applied to a bulk materials system.

### Energy Filtering Effect

**Fig. 3:**
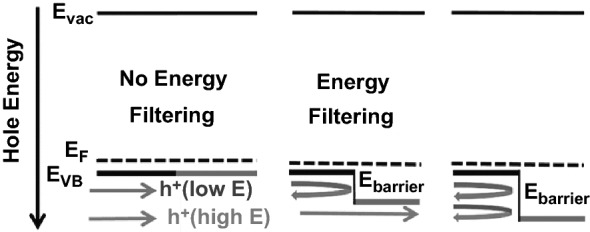
An illustration of the energy filtering effect. In the middle image, carriers (holes) with high energy (red-arrow) travel unobstructed through the energy barrier while carriers with lower energy (blue) are blocked. Adapted with permission from Ref. [60]. (Color figure online)

The first concept of energy filtering was introduced by Ioffe in 1959 [57] and further investigated by Rowe and Min in 1995 [58]. They studied the effect of different barriers on the *σ* and the *S* by the relaxation-time approximation method, indicating that the flow of minority charge carriers should become obstructed by the high-energy barriers, thereby suppressing the bipolar effects. This presented obvious reduction of the *σ* by promoting transport of the primary charge carriers. Nanostructures can also be used to enhance the *PF* via the energy filtering. The presence of nano-sized precipitates can act as a filter for carriers with low energy, therefore increasing the *S* and hence the *PF*. An illustration of the energy filtering mechanism is shown in Fig. 3. It is noteworthy that the overall benefit of the energy filtering is debatable, even to date. On one hand, by filtering out the low energy carriers, the *S* can be improved. On the other hand, the fewer number in carriers leads to a decrease in the *σ*. More recently, the manifestation of the energy filtering was elucidated in terms of the scattering exponent (*r*). The change in *r* from − 0.5 for the acoustic phonon scattering to near 0 due to the energy filtering has been shown to be greatly beneficial for enhancement in TE performance [59].

### Phonon Scattering Effect by Nanoparticles

In low dimensional or nanostructured TE materials, in addition to the phonon–phonon scattering as the dominant effect, the phonon scattering effect due to nanostructures cannot be negligible. Recently, it is generally accepted that the nanoparticles or nanocrystals can be easily *in-situ* in bulk materials to obtain the lower *κ* [8]. Here, the phonon relaxation time of nanoparticle scattering (${\tau }_{NP}$) is given by the Mathiessen-type interpolation between the short- and long-wavelength scattering regimes [61].

3 $$\frac{1}{{\tau _{{NP}} }} = \nu \left( {\sigma _{S}^{{ - 1}} + \sigma _{1}^{{ - 1}} } \right)^{{ - 1}}$$

4 $${\sigma}_{S}=2\pi {R}^{2}$$

5 $$\sigma _{1} = \pi R^{2} \frac{4}{9}\left( {\frac{{{{\Delta }}D}}{D}} \right)^{2} \left( {\frac{R}{\nu }} \right)^{4}$$

The parameter *ρ* is the density of a nanoparticle/nanocrystal, *R* is the radius of a nanoparticle/nanocrystal, *D* is the density of the matrix, and *ΔD* is the difference in densities between a nanoparticle/nanocrystal and matrix. Kim et al*.* observed an apparent reduction in the *κ* by almost a factor of 2 below the limit of alloy when embedding the ErAs nanoparticles into In_0.53_Ga_0.47_As [62]. According to the simulation of the *κ* of Si–Ge nanocomposite [63], SiGe nanoparticle-in-alloys [61], an observable decrease in the *κ* was reported. In previous studies, Kundu et al*.* noted that the decrease in the *κ* of nanoparticles materials of matrix depended on the relative atomic mass difference between the nanoparticle and matrix, which is consistent with Eq. (3) [64, 65]. Similar reduction of the *κ* was also observed in various TE materials with nanostructuring such as PbTe [66, 67], PbS [68, 69], and SnTe [70], thus improving the ultimate TE performance.

## Bottom-up Nanostructuring of Thermoelectrics

To date, the most popular method to prepare thermoelectrics is to use conventional solid-state sintering that involves ball milling and/or spark plasma sintering (SPS), which is energy-consuming and lack of mechanisms to precisely control the size, shape and surface chemistry. In comparison with the top-down nanostructuring or nanopatterning such as electron beam lithography [71], the bottom-up approaches are relatively cost-effective and also offer advantages in controlling phase purity, crystallinity, density and dimensions. Nanostructured or nanocomposite thermoelectric materials can help enhance *ZT* via increasing power factor through modulation doping, decreasing thermal conductivity via phonon scattering [72], increasing *S* by modulating the density of states of carriers, and energy filtering which results in simultaneous increase in *S* and *σ* [73]. In addition, nanomaterials may help to enhance mechanical properties via precipitation hardening which pins dislocations from moving [74]. However, the difficulties in designing nanostructured thermoelectrics lie in strong inter-correlation between materials transport properties, which demand careful adjustment of carrier densities. Therefore, tuning the size, shape, composition and phase is important in addition to distribution and orientation of these nanostructures. This is to ensure the coherence and band alignment between different phases in nanostructured TE materials, and modulate the effect of defects (dislocations, point defects, and stacking faults) and surface roughness on the thermoelectric properties such as thermal conductivity.

In terms of materials processing, vacuum-based thin film deposition methods are the only mature technologies to date that can reliably produce nanostructured materials and accurately control the composition or stoichiometry. In thin film thermoelectrics, some of the highest ever reported power factor have been reported [75]. Nevertheless, the main drawback from such vacuum deposition is its high cost and low productivity. In addition, it cannot be so easily scaled up to obtain bulk or thick films, and even not to mention the various issues in accurately measuring the thermoelectric properties, especially *S* and *κ*. During these measurements, the heat from hot side not only transfers to cold side, but also to the substrate underneath the film, which makes data interpretation extremely challenging. In comparison, nanostructuring via solution-based processes is very attractive because it does not require substrate (often made of expensive single crystal) to support the growth and can be readily scaled up. The solution-based bottom-up processes also provide convenience for the device fabrication in terms of size, shape, flexibility and conformability, which is promising for application in wearable thermoelectric energy harvest systems.

In this section, the recent progress on various bottom-up approaches towards the preparation of nanostructured thermoelectric materials with different dimensions will be summarized, their structure–property relationships as well as mechanisms for performance enhancement will also be discussed.

### 0D Nanoparticles and Nanoinclusion

#### Colloidal Synthesis

At present, vapor-phase approaches are generally found not suitable for the synthesis of high-quality nanocrystals due to existing limitations found in instruments and precursors [76–78]. In contrast, the liquid-phase colloidal synthesis of monodisperse semiconductor nanocrystals can offer a convenient route towards low-cost and scalable low-dimensional TE materials. In addition, the optoelectronic properties of nanocrystals can be tuned via synthesis, engineering surface of nanocrystals and control of the size down to sub-10 nm range. This opens up the possibility to explore properties of TE materials with strong quantum-confine effect [79]. The use of capping ligands and surfactants facilitates the dispersion of colloidal semiconductor nanocrystals in solvents. The shapes and sizes of nanocrystals can be easily tailored by making use of the kinetic control over the nucleation and growth processes with the assistance of organic ligands. The colloidal semiconductor nanocrystals exhibit attractive TE features owing to the low dimensionality of the materials. On one hand, abundant grain boundaries are able to scatter the phonons to reduce the *κ*. On the other hand, the quantum confinement effect brings about increased density of states near the Fermi level, giving rise to an enhanced *S*. Moreover, the stable colloidal suspensions are highly solution-processable, making them particularly attractive for ultrahigh throughput device manufacturing such as spin-coating, inkjet printing and roll-to-roll casting [80, 81].

Despite the above advantages, the major obstacle of exploiting solution-processed nanocrystals for high-performance TE devices is that organic ligands are insulating in nature, hindering charge transfer between nanocrystals. Furthermore, the large interface of nanocrystals adversely affects the charge transfer processes, and thus leads the *σ* of nanocrystal films to inevitably low. Therefore, the key to improve the TE performance of nanocrystals is to find an effective way to remove the organic ligands on the surface of the nanocrystals after synthesis, and properly engineer electronic coupling at the interface of nanocrystals, while maintaining the nanocrystal features such as the quantum confinement effect and interfaces.

**Fig. 4:**
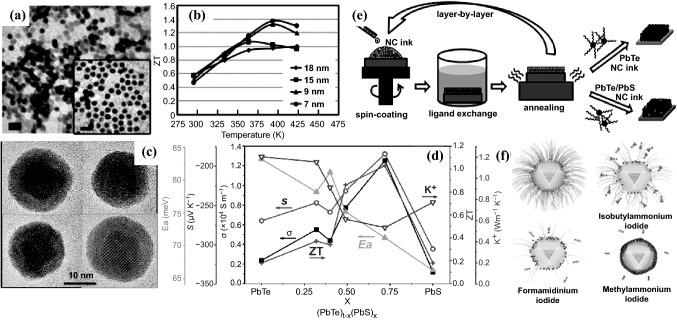
**a** TEM image of colloidal PbSe QDs after metal chalcogenide complex treatment with a diameter of 9 nm. **b** *ZT* of the prepared PbSe(SnS_2_) QD films versus temperature. **c** HRTEM graphs of a few (PbTe)_0.25_@(PbS)_0.75_ core–shell nanoparticles. **d** *σ*, *S*, porosity-corrected *κ*, and *ZT* at 710 K and activation energy for electrical transport in the low-temperature range (E_a_), as a function of the PbS concentration in the core–shell (PbTe)_1−*x*_ (PbS)_*x*_ nanoparticles. **e** Synthesis of colloidal nanocrystal thin films by layer-by-layer method and strategy for optimizing TE performance. **f** Schematic of oleic acid ligand exchange using different iodide salts. Adapted with permission from Refs. [83–86]

In 2008, Wang et al*.* prepared colloidal PbSe nanocrystals with sizes from ∼4.3 to ∼8.6 nm by reacting lead oleate with tri-n-octylphosphine selenide in squalane in the presence of oleic acid capping ligands [82]. Oleic acid at the nanocrystal surface was then stripped by hydrazine, which reduced the interparticle spacing from ∼1.1 down to 0.4 nm and resulted in a greatly improved *σ*. In 2013, Yang et al*.* synthesized PbSe colloidal quantum dots (CQD) with different sizes using conductive metal chalcogenide complexes SnS_2_–N_2_H_4_ to replace the organic ligands (Fig. 4a) [83]. It was found that the films of smaller QDs have a larger *S*, indicating the presence of stronger quantum confinement, and lower *σ* and *κ* values. The prepared PbSe(SnS_2_) QD films displayed enhanced *ZT* from 0.5 at room temperature to 1.0–1.37 at 400 K (Fig. 4b). In 2016, Ding et al*.* reported a convenient approach to fabricate spin-coated thin films with colloidal lead chalcogenide nanocrystals using a two-step interface engineering treatment (Fig. 4e): (1) the ligand exchange process was performed on the PbTe or PbTe/PbS layer by treatment with ethylenediamine, and (2) the post-treated layer was then annealed at different temperatures for proper coupling of the nanocrystals [84]. Notably, the nanocrystal thin films were found to exhibit have a higher *S* (400–460 μV K^−1^) than that of bulk PbTe, which is believed to be ascribed to the effect of the quantum confinement of the nanocrystals. The *S* of all the PbTe nanocrystal thin films was observed to decrease with increasing annealing temperature, likely due to the weakened quantum confinement effect of the fused nanocrystals. It was also found that ligand replacement with ethylenediamine could assist in necking between nanocrystals, resulting in an increased σ. The fabricated PbTe/PbS nanocrystal thin films exhibited a high ZT value of ≈ 0.30 at 405 K after thermal annealing at 400 °C.

The replacement of short-chain organic ligands to modify colloidal nanocrystals leads to strong inter-particle electronic coupling and thus promotes efficient charge transport in colloidal nanocrystals films. In addition to offering excellent surface defect passivation, the use of appropriate halides in engineering the electronic coupling in nanocrystal films would be also crucial for efficient charge transport. In 2019, Nugraha et al*.* reported n-type TE iodide-capped PbS CQD film which allows for the fabrication of highly efficient TEG devices [85]. The counter-ions in iodide salts were found to play a critical role in facilitating ligand removal and charge transport in CQD films (Fig. 4f). Methylammonium iodide (MAI) could bring about efficient charge transport in the QD films which was resulted from the complete removal of oleic acid ligands and excellent passivation of surface defects. An impressive improvement in the *σ* of 100%, exceeding 12 S cm^−1^, was obtained for the MAI-treated CQD films, leading to a promising n-type *PF* of up to 24 Μw m^−1^ K^−2^ at relatively low temperatures (< 360 K), which was significantly improved compared to previously reported n-type lead chalcogenide CQD films (< 1 μW m^−1^ K^−2^).

In 2013, Ibanez et al.reported a one-pot two-step colloidal synthetic route to prepare PbTe@PbS core–shell structured nanoparticles with narrow size distributions and exceptional composition control (Fig. 4c, d) [86]. As-synthesized PbTe@PbS nanoparticles served as the building blocks for bottom-up production of PbTe–PbS nanocomposites. Interestingly, a doping-like effect was observed when PbTe and PbS were mixed at the nanometer scale. In such PbTe–PbS nanocomposites, synergistic nanocrystal doping effects resulted in up to tenfold increase in the *σ* compared to pure PbTe and PbS nanomaterials alone. Without intentionally doping of any of the two phases, (PbTe)_0.28_(PbS)_0.72_ reached *σ* up to 1.2 × 10^4^ S m^−1^. At the same time, the acoustic impedance mismatch between PbTe and PbS phases and a partial phase alloying collectively provided PbTe–PbS nanocomposites with a significantly reduced *κ* (down to 0.53 W m^−1^ k^−1^). As a result, a high TE ZT of ∼1.1 was obtained at 710 K.

#### Hydrothermal/Solvothermal Synthesis

Hydrothermal synthesis involves the growth of crystals with different sizes at the submicron to nanometer scale. This is usually achieved via chemical reactions in an aqueous medium, at elevated temperature and high pressure. The successful synthesis of the nanostructures is highly dependent on the precise control over the internal reaction conditions, such as reaction time, pressure, pH value, reagent concentration, and presence of organic additives or templates, as well as external reaction environment including microwave or conventional heating. The solvothermal method can be employed to have more control over the size, shape, reactivity, and phase of the nanostructures in organic solvents than in water. The viscosity and polarity of solvent can influence the transport behavior and solubility of the reagents in the liquid medium, and hence the properties of nanostructured product. Although there are many reports on the preparation of semiconductor nanocrystals using hydrothermal/solvothermal methods [87–90], these approaches generally require high temperature and pressure as well as prolonged reaction periods, which significantly hinder applications for large-scale synthesis.

**Fig. 5:**
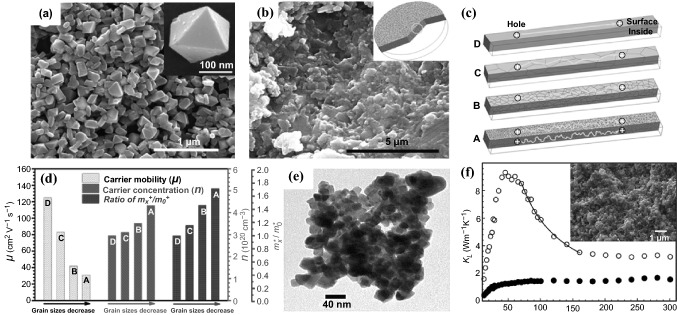
**a** SEM image of the SnTe nanoparticles synthesized by microwave hydrothermal method. **b** SEM image of the dense specimen from the 165 nm nanoparticles. **c** Proposed electrical transporting mechanism of the dense samples sintered from the 165 nm, 550 nm nanoparticles (NPs), 8.2 μm microparticles (MPs), and mechanically alloyed (MA) powder. **d** Hole mobility, carrier concentration and the ratio of *m*_x_*/m_0_* as function of the decreased grain sizes. **e** TEM image of 40 nm FeSb_2_ nanocrystals prepared by solvothermal method. **f** κ_L_ of the densified FeSb_2_ nanocrystals (solid circle) and bulk FeSb_2_ (open circle). The line of 1/T dependence (solid line) is indicative of phonon–phonon scattering. The inset shows an SEM image of a fractured surface of the densified FeSb_2_ specimen. Adapted with permission from Refs. [91–93]

In 2016, Li et al*.* reported a facile, rapid, environmentally-green and high-yield microwave hydrothermal method for preparing SnTe nanoparticles with controlled sizes from micro-scale to nano-scale [91]. The reaction rates can be controlled by adjusting the concentration of reagents, resulting in SnTe nanoparticles with sizes ranging from 165 to 8.2 nm. After the SPS treatment, an ultra-low *κ* of 0.60 W m^−1^ K^−1^ at 800 K, being only 11.8% of the reference sample, was obtained using the 165 nm nanoparticles (Fig. 5a) owing to the enhanced phonon scattering effect introduced by refined grains, grain boundaries and point defects in the sintered dense materials. This sintered materials (Fig. 5b) exhibited a relatively higher *S* (58–90 μV K^−1^, 323–800 K) and much a higher *ZT* value (about 0.49 at 803 K) compared to pure SnTe bulk material, which can be ascribed to the enhanced phonon scattering and the intensified energy filtering effect. The size effect was also found to have some influences on the *σ*. The electronic transport mechanism (Fig. 5c) was proposed and the hole mobility, carrier concentration and the effective mass (*m*_x_*/*m*_0_*) as function of the decreased grain sizes were measured by the corresponding carrier mobility test as shown in Fig. 5d. Evidently, the hole mobility reduced from 125 to 31 cm^2^ V^−1^ s^−1^ while the carrier concentration increased when the grain sizes gradually decreased to 165 nm with the increasing crystal defects. It is noteworthy that the decrease in mobility is higher than the increase in carrier concentration, which results in a lower *σ*. In 2017, Tang et al*.* reported the synthesis of tetradecahedron Cu_2_S microcrystals with the size of 1–7 μm via the hydrothermal method, which could achieve a *ZT* value of 0.38 at 573 K after the SPS process [92]. Datta et al*.* also developed phase-pure FeSb_2_ nanocrystals with an average size of 40 nm (Fig. 5e) through an ethanol-mediated, low-temperature solvothermal process [93]. Without a template or capping chemical used in the process, these nanocrystals grew in their inherent orthorhombic symmetry. Due to significant grain boundary phonon scattering, the densified FeSb_2_ nanocomposite showed a drastic reduction in *κ*_L_ compared to the bulk material prepared by a solid-state synthesis process (Fig. 5f).

#### Electrodeposition

In addition to the above described methods, electrodeposition is another unique process known to make nanoparticles with a controlled size and morphology [94, 95], offering the benefits of fast speed, simplicity, low-cost and avoidance of use of binders. Moreover, the electrochemical deposition of TE materials enables the easy fabrication of thin TE film although this method still suffers from drawbacks, such as the existence of impurity in as-deposited thin films and poor crystallization. Therefore, it is imperative to precisely control the composition and crystallographic structure of nanoparticles to achieve an effective electrodeposition process.

**Fig. 6:**
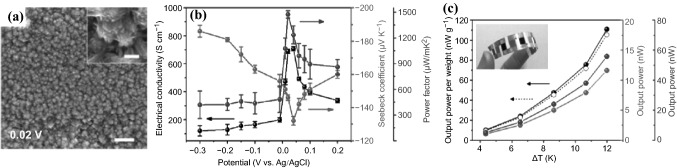
**a** SEM image of electrodeposited Bi_2_Te_3_ nanocrystals at a potential of 0.02 V (scale bar: 500 nm). **b** Corresponding σ (black circle), S (red circle), and PF (blue circle) of the flexible TE generators (FTEGs) with Bi_2_Te_3_ films deposited at different potentials (V vs. Ag/AgCl). c Output power per weight of 1-couple (filled black circle) and 5-couples (open black circle) and output power of 1-couple (red circle) and 5-couples (blue circle) of the pn-FTEGs over a temperature gradient (ΔT). Adapted with permission from Ref. [96]. (Color figure online)

In 2016, Na et al*.* reported a method for preparing highly conductive n-type Bi_2_Te_3_ nanocrystal films on a flexible substrate using electrodeposition [96]. The growth of the Bi_2_Te_3_ crystals was precisely controlled by adjusting the electrochemical deposition potential, which was critical to modulate the size and preferential orientation of the crystal growth along the (110) direction, and thus to improve the TE properties of the fabricated flexible TE generator (FTEG) (Fig. 6b). A Bi_2_Te_3_ nanocrystal film prepared under a potential of 0.02 V (Fig. 6a) showed a high *σ* (691 S cm^−1^) with a maximum *PF* of 1473 μW m^−1^ K^−2^, which is the highest among the Bi_2_Te_3_ films prepared by the electrodeposition methods. Integrating it with an n-type Bi_2_Te_3_ FTEG, a prototype of a p-n-type flexible TEG (pn-FTEG) was prepared using a p-type polymer, poly(3,4-ethylenedioxythiophene)s. The pn-FTEG (5-couples) generated an output voltage of 5 mV at ΔT = 12 K with a high output power of 105 nW g^−1^ (Fig. 6c). Very recently, Zhao et al*.* also prepared micrometer-thick Bi_2_Te_3_ nanocrystal films using the electrochemical deposition process [97]. The optimum *PF* of as-grown Bi_2_Te_3_ films was achieved by shortening the period of the electrochemical deposition and introducing a photon-based rapid annealing process for the material post-crystallization. Compared with single crystalline or vacuum deposited Bi_2_Te_3_ films, the electronic transportations of the electrochemically deposited Bi_2_Te_3_ are more influenced by the carrier scatterings by the grain boundaries and lattice defect. In 2019, Nguyen et al*.* reported the synthesis of the gold nano-particles-bismuth telluride composites using the electrochemical co-deposition, and significant improvement in TE properties was achieved [98]. The composite with 5 wt% of 5 nm-diameter gold nanoparticles showed a highest absolute *S* of ∼380 μV K^−1^, a low *κ* of ∼0.5 W m^−1^ K^−1^ and a high ZT of ∼0.62 at room temperature.

#### 0D-Nanoinclusion

The enhancement in TE properties of nanostructures by lowering the *κ* is often off-set by the concurrent deterioration of the *σ*. As such, selectively lowering the *κ* without compromising with the *σ* remains a challenge. One possible way is to embed nanoparticles (particularly metal nanoparticles) with controlled sizes into a bulk matrix to increase *ZT* values. The advantage of incorporating nanoparticles into TE materials is able to reduce the *κ*_L_ due to the interfaces scattering of heat-carrying phonons, and it simultaneously enhances the *S* via the electron energy filtering effect caused by the scattering of electrons on the band bending at the interfaces between nanoinclusions and the semiconductor host. The enhanced *S* could compensate for the reduction in the *σ* to some extent, thus maintaining the *PF* at the similar level. The nanoinclusion composite structures have been generally prepared by the ball milling of metal (Ag, Au, Cu, Zn) or ceramic (ZrO_2_, SiC) nanoparticles with TE raw materials as the matrix phase [99–106]. However, such top-down processes for fabricating the low-dimensional structures are relatively expensive and time-consuming, probably not feasible for large-scale synthesis. Furthermore, enhancement in TE performance may be limited by the inhomogeneous distribution and aggregation of nanoparticles within the matrix.

In 2014, Sun and co-workers demonstrated the first bottom-up preparation of textured n-type Bi_2_Te_2.7_Se_0.3_ thin films with content-adjustable Pt nanoinclusions by the pulsed laser deposition [107]. Addition of Pt nanoinclusions resulted in a higher in-plane *PF* based on the simultaneous increase in both the *σ* and absolute *S*. The *PF* of the optimized nanocomposite thin film reached 3.51 × 10^–3^ W m^−1^ K^−2^ at room temperature, which is a more than 20% enhancement as compared to the single phase Bi_2_Te_2.7_ Se_0.3_ thin film.

**Fig. 7:**
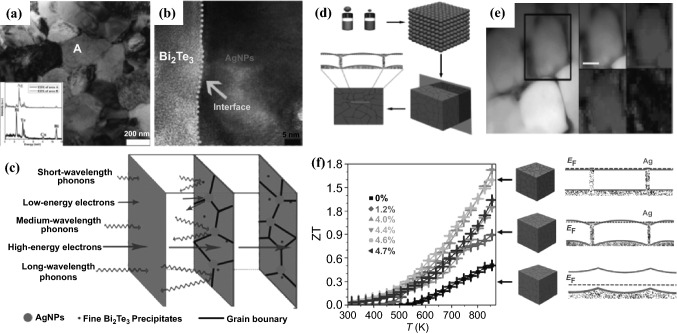
**a** Low magnification TEM image of the bulk Bi_2_Te_3_ nanocomposite with 5.0 vol% AgNPs nanocomposites. **b** HRTEM image showing the newly-built interface between the Bi_2_ Te_3_ matrix and AgNPs. **c** Schematic drawing of a hierarchical two-phased heterostructure, showing the strong scattering of phonons with multiscale mean free paths along with the energy filtering effect. **d** Bottom-up assembly process to produce PbS–Ag TE nanocomposites from the assembly of PbS (blue) and Ag (red) NCs, and the corresponding band alignment of the resulting nanocomposite. **e** HRTEM and elemental EDX mapping of the PbS–Ag 4.4 mol% nanocomposite. **f** Figure of merit and schematic representation of the electron energy band alignment in PbS–Ag nanocomposite. Adapted with permission from Refs. [108, 109]. (Color figure online)

In 2015, Zhang et al*.* developed a facile and robust bottom-up chemical route to synthesize silver nanoparticles (AgNPs)-dispersed Bi_2_Te_3_ composites with a hierarchical two-phased heterostructure, in which the Bi_2_Te_3_ nanopowder was prepared by the surfactant-mediated hydrothermal method and AgNPs (60 nm) were obtained by using polyol reduction of silver nitrate, respectively, followed by the ultrasonic dispersion treatment and the SPS process [108]. The results clearly demonstrated that uniformly-dispersed AgNPs could lead to (1) growth-suppression of Bi_2_Te_3_ grains, (2) the introduction of nanoscale precipitates, and (3) the formation of new interfaces with Bi_2_Te_3_ matrix, leading to a hierarchical two-phased hetero-structure (Fig. 7a, b), which caused the intense scattering of phonons with multiscale mean free paths (Fig. 7c) and therefore significantly reduced the *κ*_L_. Meanwhile the improved *PF* is maintained because of the Ag’s low-energy electron filtering and superior electrical transport. A maximum *ZT* value of 0.77 was obtained at 475 K from the bulk Bi_2_Te_3_ dispersed with 2.0 vol% AgNPs, which was significantly enhanced by 304% compared with that of the pristine bulk Bi_2_Te_3_.

In 2016, Ibanez reported the preparation of consolidated yet nanostructured TE materials based on a straightforward and versatile strategy involving bottom-up assembly of colloidal nanocrystals (Fig. 7d) [109]. PbS–Ag nanocomposites were prepared by mixing cubic PbS nanocrystals (ca. 11 nm) with spherical Ag nanocrystals (ca. 3 nm), followed by the removal of solvent through evaporation. Annealing was then performed to remove residual organic compounds, after which the resultant powdered nanocrystal blend was hot-pressed into pellets. PbS-Ag nanocomposites showed a highly homogeneous distribution of Ag nanodomains at the interfaces of PbS grains, as evidenced by high-resolution transmission electron microscope (HRTEM) (Fig. 7e). Ag nanodomains in the nanocomposites not only blocked phonon propagation, but also supplied electrons to the PbS host semiconductor and reduced the energy barriers between PbS crystal domains (Fig. 7f). The resultant nanocomposites therefore exhibited a reduced *κ* and a higher charge carrier concentration and mobility compared to pure PbS nanomaterial. The *σ* of the composites can reach up to 660 S cm^−1^ with a Ag concentration of above 4 mol%. The simultaneous combination of an outstanding *σ*, a relatively large *S*, and a reduced *κ* contributed to a *ZT* up to 1.7 at 850 K.

Apart from the highly conductive metal nanoinclusions [110, 111], Lim *et.al.* has recently introduced nonmetal (Te or Se) nanodomains into a silver selenide matrix through solution blending of Ag_2_Se nanoparticles with Te or Se nanorods before powder consolidation [112]. Different from the injection of conductive metal nanoinclusions that can lead to both an enhanced *σ* and a lower *S*, the injection of a reduced concentration of charge carriers into a doped semiconductor could cause a band bending that promotes electron filtering. Interfacial energy filtering effects resulted in remarkable improvement in the *S* being recorded for nanocomposite with 5 wt% Te nanoinclusion without significantly compromising with the *σ*. This nanocomposite displayed an improved average *ZT* value of 0.84 in the temperature range of 300–400 K, higher than most of previously reported bulk Ag_2_Se.

### 1D Nanowires/Nanofibers/Nanotubes

The *ZT* values of TE materials can be improved by introducing 1D nanostructures by (1) increasing *PF* through the quantum confinement and/or energy filtering effect, or (2) reducing the *κ*_L_ via the enhanced phonon scattering [80, 113, 114]. The reduction in dimensionality from 3D bulks to 1D results in an enhancement in the electronic density of states at the energy band edges and thus causes an increase in the *PF*. In this section, we will introduce the bottom-up methods for preparation of semiconductor NWs and their structure–property relationships will be summarized.

#### Solution-phase Synthesis

**Fig. 8:**
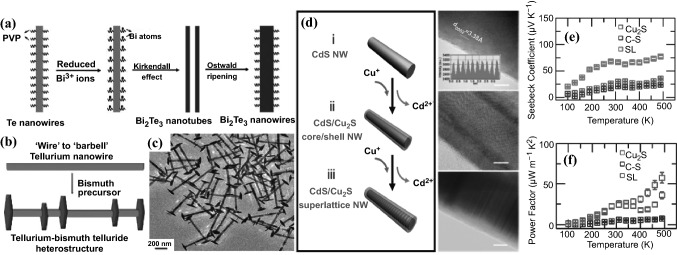
**a** Schematic illustrations of the formation mechanism of the Bi_2_Te_3_ NWs via a template-assisted solution phase process. **b** Schematic of tellurium NW formed in the first step and tellurium–bismuth telluride heterostructure after adding bismuth precursor in the second step. **c** TEM image of the Te–Bi_2_Te_3_ heterostructured NWs. **d** Schematic diagram and corresponding TEM images (scale bar: 5 nm) of phase evolution from pristine CdS NW to CdS/Cu_2_S core–shell NW, and eventually to CdS/Cu_2_S superlattice NW. Temperature dependence of **e** thermopower and **f** power factor of Cu_2_S nanowire, CdS/Cu_2_S core–shell nanowires (C–S) and CdS/Cu_2_S superlattice nanowires (SL). Adapted with permission from Refs. [115, 120, 121]

Over the past decade, solution phase template-directed synthesis has been widely employed for the preparation of 1D metal chalcogenide NWs. In 2011 Wang et al*.* first reported a synthetic method for the controlled formation of ultrathin Te NWs using polyvinylpyrrolidone (PVP) surfactant. The as-prepared Te NWs served as sacrificial templates to facilitate the synthesis of highly uniform Bi_2_Te_3_ NWs with a diameter of 15–17 nm and a length of tens of micrometers. The reaction was performed in triethylene glycol solution by adding Bi precursor and hydrazine to Te NWs at 200 °C and atmospheric pressure [115]. The formation of Bi_2_Te_3_ NWs was demonstrated to be a result of the Kirkendall effect and Ostwald ripening (Fig. 8a). Using a similar method, Zhang et al*.* also prepared n-type ultrathin Bi_2_Te_3_ NWs with an average diameter of 8 nm in ethylene glycol solution with a high yield up to 93% [116]. The as-prepared Bi_2_Te_3_ NWs were then compressed to bulk pellets via the SPS process and was found to exhibit a high *ZT* value of 0.96 at 380 K, attributing to the increased phonon scattering taking place at NW boundaries and hence resulting in a notable decrease in the *κ*. Following this method, different kinds of binary telluride NWs with small diameters, including PbTe [117], CdTe [118], Cu_2_Te [119], and Ag_2_Te NWs [117], have been prepared via a solution phase synthesis. Solution phase synthesis can therefore be potentially translatable for low-cost, large-scale synthesis of materials for TE applications.

Compared with binary alloys, ternary alloys exhibit greater potential in tuning the band gap, elemental composition, charge-carrier density and conductivity, making them more promising for TE materials. In 2016, Zhou et al*.* reported a general synthesis approach to prepare ultrathin ternary metal chalcogenide NWs [122] involving the synthesis of ultrathin 6 nm diameter Te NWs, which served as precursors to fabricate Te_*x*_Se_1−*x*_ NWs with tunable aspect ratios. The as-prepared Te_*x*_Se_1−*x*_ NWs could then be converted into a series of ternary alloyed metal-Te_*x*_Se_1−*x*_ NWs by injecting appropriate metal precursors under specific conditions. A series of ultrathin ternary alloyed NWs, including Bi_2_Te_*x*_Se_3−*x*_, Ag_2_Te_*x*_Se_1−*x*_, Cu_1.75_Te_*x*_Se_1−*x*_, CdTe_*x*_Se_1−*x*_, and PbTe_*x*_Se_1−*x*_ were synthesized and characterized for the first time, with controllable Te/Se ratios. Among these, Bi_2_Te_2.7_Se_0.3_ NW-based bulk material exhibited outstanding TE performance with a high *PF* of 1023 μW m^−1^ K^−2^ and a *ZT* of 0.75 at 320 K.

In 2012, Wu’s group demonstrated a design principle to prepare new categories of telluride-based TE NW heterostructures through solution-phase reactions [120]. The catalyst-free synthesis started with the preparation of Te NWs, followed by the growth of Bi_2_Te_3_ nanoplates on the Te NW tips and bodies, yielding Te–Bi_2_Te_3_ “barbell” NW heterostructures with a narrow diameter (~ 36 nm) and length distribution as well as a rough control over the density of the hexagonal Bi_2_Te_3_ nanoplates by varying the reaction conditions (Fig. 8b, c). The hot-pressed nanostructured bulk pellets of the Te–Bi_2_Te_3_ heterostructure showed a largely enhanced *S* (up to 608 μV K^−1^ at 300 K) and a greatly reduced *κ* (0.365 W m^−1^ K^−1^ at 300 K) due to the energy filtering effect occurring at the grain–grain interfaces and the phonon scattering at the NW–NW, NW–plate, and plate–plate interfaces. In the follow-up study, these “barbell”-like Te–Bi_2_Te_3_ NWs were further converted to other telluride-based compositional modulated NW heterostructures such as PbTe–Bi_2_Te_3_ and Ag_2_Te–Bi_2_Te_3_, which displayed a high *ZT* of 1.2 (at 620 K) and 0.41 (at 400 K), respectively [123, 124].

A low-cost solution process, the strain induced selective phase segregation technique, to produce superlattice nanostructures, was reported by Tang, et al*.* in a CdS/Cu_2_S system. Due to the energy filtering effect, the superlattice NWs exhibited an improved *S* without sacrificing the *σ* [121]. The distinct interface formation energy at different CdS facets and the self-regulated strain energy relaxation at the CdS–Cu_2_S interface facilitated the conversion of CdS NW into CdS/Cu_2_S core–shell structures (Fig. 8d). Their *S* can be significantly enhanced (Fig. 8e) by the energy filtering effect which was favored by the junction formed at the CdS–Cu_2_S interface, while the *σ* of the superlattice NWs was not greatly compromised, leading to greatly enhanced power factor at temperature higher than 400 K (Fig. 8f).

#### Vapor–liquid–solid Growth

**Fig. 9:**
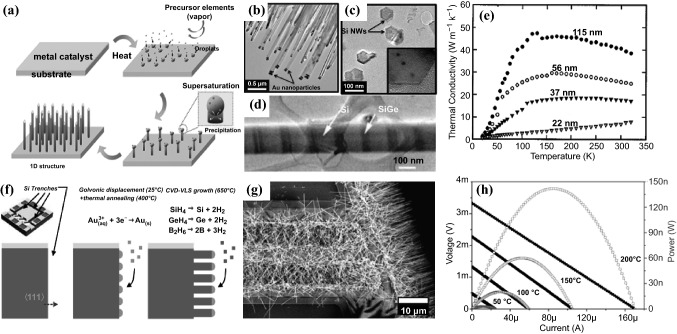
**a** Schematic diagram of the synthesis of the NWs by VLS growth method. **b** TEM image of Si NW arrays showing gold nanoparticles on the tip. **c** Cross-section TEM image of silicon NWs showing hexagonal shape. **d** TEM image of Si/SiGe superlattice NWs. **e** Measured thermal conductivity of different diameter Si NWs. **f** Schematic steps for CVD-VLS growth of boron-doped SiGe NWs and integration in μTEGs. **g** Top-view SEM image of SiGe NWs selectively grown in Si exposed parts. **h** Current–voltage and current-power curves obtained from SiGe NWs based μTEGs with three trenches for different hot plate temperatures. Adapted with permission from Refs. [127–129]

The vapor–liquid–solid method (VLS) is widely used to grow 1D structures, such as NWs [125, 126]. The synthetic procedures typically start with the deposition of metal catalyst on a substrate, which is then converted to liquid alloy droplets by adsorbing the precursor vapor component at a high temperature (Fig. 9a). Crystal growth through direct gas phase adsorption onto a solid surface is typically very slow. The VLS method therefore circumvents this by introducing a catalytic liquid alloy phase which can rapidly adsorb a vapor to a supersaturation level, allowing crystal growth to occur from nucleated seeds at the liquid–solid interface. The bottom-up growth of semiconductor NWs by the VLS method is able to precisely control their size, morphology, growth density, spatial distribution, composition as well as element doping.

In an early work, Li et al*.* fabricated VLS-grown individual single crystalline intrinsic Si NWs with diameters of 22, 37, 56, and 115 nm [127]. The size effects on the *κ* of these individual NWs were studied (Fig. 9e). The *κ* observed was lower than the bulk value, and the strong diameter dependence of the *κ* in NWs was ascribed to the increased phonon-boundary scattering and possible phonon spectrum modification. Using the hybrid pulsed laser ablation and the VLS growth process, the same group also prepared single crystalline Si/SiGe superlattice NWs (Fig. 9d) with diameters of 58 and 83 nm [128]. Compared with the pure Si NWs in which alloy scattering is suggested to be the dominant phonon scattering mechanism for the thermal transport, this study demonstrated that the NW boundary scattering played a role in reducing the *κ*. In 2011 and 2012, Kim and Park et al*.* synthesized VLS-grown rough Si and Si_0.96_Ge_0.04_ NWs with various surface roughness and diameters with the assistance of different catalysts [130, 131]. It was found that the surface roughness affected the *κ* more significantly than the diameter of the NWs. Theoretical analysis reveals that the surface roughness scattering affects mid-wavelength phonons, whereas the phonon boundary scattering affects long-wavelength phonons and the alloy scattering affects short-wavelength phonons.

The characteristics of CVD-VLS process enables the convenient creation of microscale TE devices with control over photonic, electronic, and thermal properties. In 2013, Davila et al*.* first fabricated dense arrays of well-oriented and size-controlled Si NWs (Fig. 9b, c) obtained from the CVD-VLS process and implemented them into microfabricated structures to make a planar unileg TE microgenerator (uTEGs) [132, 133]. The average diameter and the length of the Si NWs are 100 nm and 10 μm, respectively. The resulting TEG can generate power densities of 1.44 mW cm^−2^ and 9 μW cm^−2^ under temperature differences of 300 and 27 K, respectively. In 2017, Hill et al. synthesized VLS-grown uniform, linear, and degenerately boron- and phosphorous-doped Si NW superlattices with abrupt transitions between p-type, intrinsic, and n-type segments [134]. Recently, Noyan et al*.* reported the bottom–up growth of SiGe NW arrays by means of CVD-VLS and their monolithic integration into TE microgenerators (Fig. 9f–h) [129]. Densely aligned boron-doped SiGe NWs with a diameter of 64 ± 11 nm, a length of 10 μm, 30% Ge content, and doping of ~ 10^20^ cm^−3^ were grown simultaneously and integrated via the gold-catalyzed CVD-VLS approach in devices with different numbers of micro-trenches. A three-trench single thermocouple placed on a 200 °C heat source could achieve a maximum power of 142 nW which is equivalent to a power density of 7.1 μW cm^−2^, demonstrating the great potential of the as-prepared material for energy harvesting from waste heat.

#### Template-Assisted Electrodeposition Method

Template-assisted electrodeposition is the most convenient method for synthesizing NWs with controlled stoichiometry, size, morphology and crystallinity [135]. Moreover, it is also cost-effective and scalable for applications. For conventional TE materials based on chalcogenide semiconductors, electrodeposition methods are widely employed for creating high-aspect-ratio NWs using hard templates with enormous 1D nanochannels. The two most common templates to obtain NWs by electrodeposition are anodic aluminum oxide (AAO) and polycarbonate (PC) membranes with different pore sizes (ranging from tens of nanometers to hundreds of nanometers) and template thickness. The AAO membranes are typically removed by chemical dissolution, while the PC membranes can be removed by either chemical dissolution or heat treatment in the air. The electrodeposition process can be conducted at three different modes: constant potential, current density and pulsed electrodeposition, among which the pulsed electrodeposition can provide more uniform growth and higher crystallinity of NWs [136].

**Fig. 10:**
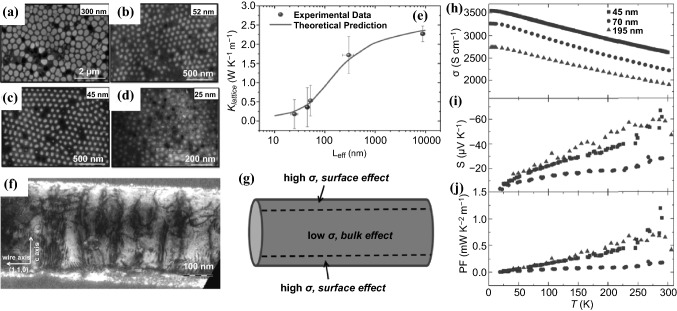
SEM images of **a** 300 nm, **b** 52 nm, **c** 45 nm and **d** 25 nm average diameter Bi_2_Te_3_ NWs. **e** κ_L_ of the NWs versus diameters. **f** TEM image of a Bi_2_Te_3−*y*_Se_*y*_ ternary NW. **g** Schematic of the NW showing the contribution of the surface states with a high *σ*, whereas bulk part yields a low electrical conductivity and a high *S*. Temperature-dependent **h** electrical conductivity, **i** Seebeck coefficient and **j** power factor of Bi_2_Te_3−*y*_Se_*y*_ ternary NWs of three different diameters (45, 70 and 195 nm). Adapted with permission from Refs. [137, 147]

It is well known that Bi_2_Te_3_ can behave as an n-type or p-type semiconductor depending on its stoichiometry. The Bi-rich composition shows a p-type semiconductor with a positive *S*, while the Te-rich stoichiometric ratio is an n-type semiconductor with a negative *S*. During the past decade, tremendous efforts have been made to prepare Bi-Te NWs with tunable compositions, morphologies and crystallographic structures using the template-assisted electrodeposition method [137–144]. In 2017, Proenca and co-workers studied the effect of deposition applied potential on the morphology, stoichiometry and crystallinity of Bi-Te NWs using the AAO template [138]. The morphology and the Te% content was found to be highly dependent on the deposition potential. X-ray diffraction measurements revealed that there was strong relationship between the material's crystallinity and the deposition potential, being monocrystalline at very low potentials, but almost completely amorphous at high potentials due to high growth rates. In the same year, Rojo et al. reported electrodeposited Bi_2_Te_3_ NWs with 300, 52, 45, and 25 nm diameters using the AAO template (Fig. 10a–d), and in-depth study of how the *κ* of Bi_2_Te_3_ NWs was affected when reducing its diameter from an experimental and theoretical point of view [145]. The *κ* was observed to decrease more than 70% (from 1.78 ± 0.46 to 0.52 ± 0.35 W K^−1^ m^−1^) when the diameter of the NW was reduced one order of magnitude (from 300 to 25 nm) (Fig. 10e). An increment of the phonon scattering is believed to be responsible for the reduction in the NWs’ *κ*. As mentioned by the Kinetic–Collective model (KCM) [146], the reduction in the *κ* is mainly caused by the alteration of the mean free path of the acoustic phonons due to the size confinement. Reeves et al*.* fabricated electrodeposited sub-10 nm Bi_2_Te_3_ NW arrays using novel silica-coated AAO templates for the pore confinement [137]. The obtained sub-10 nm NWs displayed a greatly increased electrical-to-thermal conductivity ratio as the pore diameter decreased.

Furthermore, it is well-known that Bi_2_Te_3_ can switch its n-type or p-type semiconductor response if it is doped with Se and Sb, respectively. Various ternary NWs based on Sb-doped Bi-Te [136, 148–155] and Se-doped Bi-Te NWs [147, 149, 156, 157] have also been developed using the electrodeposition method. In 2013, Baßler et al*.* published p- and n-type single-crystalline NWs of bismuth antimony telluride and bismuth telluride selenide grown by the template-based millisecond pulsed electrochemical deposition in self-ordered Al_2_O_3_ membrane templates [149]. The as-grown NWs with a diameter of 80 and 200 nm were annealed in helium and tellurium atmosphere to reduce the crystal defects which led to higher TE performance. The *PFs* of the obtained Bi_38_Te_55_Se_7_ and Bi_15_Sb_29_Te_56_ NWs reached 2820 and 1750 μW K^−2^ m^−1^, respectively, at room temperature, which are significantly higher compared to thin films as a result of the higher *σ* of the 1D structure. In 2015, Li et al*.* reported the pulse-deposited of Bi-Sb–Te NWs which displayed more homogeneous element distribution and higher crystallinity compared to direct current (DC)-deposited NWs [136]. The *ZT* of the pulse-deposited single Bi_0.5_Sb_1.5_Te_3_ NWs reached as high as 1.14 at 330 K, which is approximately 54% higher than that of the DC-deposited ones. Kumar et al.also prepared single-crystalline, ternary n-type Bi-Te-Se NWs (Fig. 10f) with different nominal diameters of 45, 70, and 195 nm by electrodeposition in a nanostructured Al_2_O_3_ matrix [147]. The transport properties of individual NWs were measured, yielding the largest *σ* (2620 S cm^−1^ at room temperature) for the smallest NW (Fig. 10h). This behavior of the *σ* might be attributed to the highly conductive surface states (Fig. 10g). Compared to bulk materials, relatively lower *S* (up to -60 μV K^−1^ at room temperature) was obtained for these NWs due to the existence of metallic surface states (Fig. 10h). The highest *PF* (0.8 mW K^−2^ m^−1^) was achieved with the 195 nm NWs (Fig. 10i), which is suppressed compared to bulk values but higher than those of thin films.

#### Electrospinning

Electrospinning is a simple and versatile technique for producing continuous nanofibers from polymers and ceramics under a high electric field with controllable morphology, diameter, composition and orientation [158, 159]. In some early work, TE properties of oxide materials have been significantly improved when their bulk 3D dimensions are reduced to 1D nanoscale by the sol–gel based electrospinning. In 2010, Yin et al*.* fabricated nanocrystalline Ca_3_Co_4_O_9_ nanofibers with diameters around 350 nm using the sol–gel electrospinning process, which were consolidated into bulk ceramics by the SPS process [160]. The nanofiber-sintered ceramic with a much smaller grain size exhibited simultaneously enhanced *S*, *σ* and thermal resistivity, resulting in 55% enhancement in *ZT* (around 0.40 at 975 K). In the same year, Ma et al*.* also reported TE nanocrystalline electrospun NaCo_2_O_4_ nanofibers with a grain size of as small as 10 nm [161], and Xu et al*.* produced TE La_0.95_Sr_0.05_CoO_3_ nanofibers with a diameter of ∼35 nm by electrospinning with a greatly enhanced *S* of 650 μV K^−1^ at room temperature [162].

**Fig. 11:**
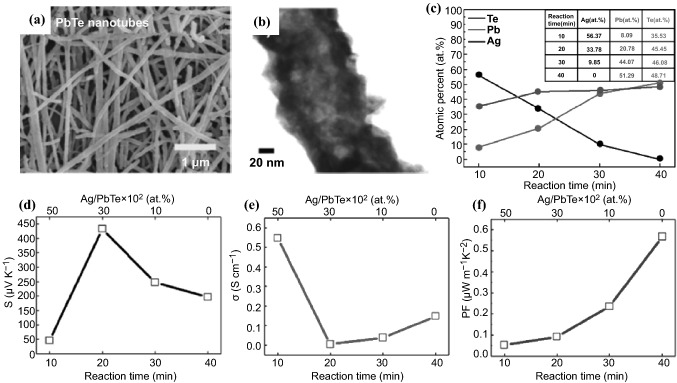
**a** SEM and **b** TEM images of PbTe nanotubes fabricated via combined electrospinning, electrodeposition and cationic exchange reaction. **c** Stoichiometry ratio of different cation exchange reaction time from Ag_2_Te to PbTe. Different reaction time of **d** Seebeck coefficient*,* **e** electrical conductivity and **f** power factor of PbTe, included 10–50% of Ag content in the 1D Ag_*x*_Te_*y*_–PbTe composite nanotubes. Adapted with permission from Ref. [163]

Metal chalcongenide semiconductor nanofibers have also been synthesized using the electropinning technique combined with electrochemical reactions. In 2018, Park et al*.* first reported the large scale fabrication of a few millimeter-long lead telluride (PbTe) hollow nanofibers employing a three-step sequential process involving electrospinning, then electrodeposition and finally, cationic exchange reaction [163]. Electrodeposition of Te onto as-prepared electrospun Ag nanofibers possessing an ultra-long aspect ratio of 10,000 afforded silver telluride nanotubes, which then underwent a cationic exchange reaction in Pb(NO_3_)_2_ solution to obtain polycrystalline PbTe nanotubes with 100 nm average diameter and 20 nm wall thickness (Fig. 11a, b). The Ag-to-Pb ratio in the Ag_*x*_Te_*y*_–PbTe nanocomposites could be easily tuned during the cationic exchange reaction (Fig. 11c), which rendered good control over the TE properties of resulting 1D hollow nanofibers (Fig. 11d–f). The content of Ag ion led to the enhancement of TE properties in the Ag_*x*_Te_*y*_–PbTe 1D nanocomposite mats, which showed the highest value of *S* of 433 μV K^−1^ at 300 K when the remained Ag content was 30%. Zhang et al*.* also fabricated PbTe hollow nanofiber mats through combining electrospinning, followed by galvanic displacement reactions using electrospun cobalt nanofibers as the sacrificial material [164]. Through tuning the diameter of the sacrificial cobalt nanofibers as well as the electrolyte concentrations in the galvanic displacement reactions, PbTe hollow nanofibers with various dimensions, surface morphologies and compositions were synthesized, demonstrating that both the quantum confinement and surface scattering effects displayed an additional degree of control over the TE properties within such polycrystalline tubular nanostructures.

### 2D Nanoflake/Nanosheet/Nanoplate

Since the discovery of graphene through the mechanical exfoliation by scotch tape in 2004 [165], various structures of 2D materials have been designed and fabricated to facilitate its physical and electronic properties because of its unique and advantageous structural features. Unlike bulk materials, 2D structure can easily tune its charge concentration, carrier transition through its network or different layers although some parameters, especially the size and shape of 2D materials, are rather difficult to be controlled [166–168]. In order to prepare various 2D structures based on semiconducting materials, various top-down methods have been designed including exfoliation, ball milling [169, 170]. This section will provide an overview of various bottom-up approaches developed recently for preparation of semiconducting 2D nanomaterials for TE applications, including vacuum-based techniques and wet-chemical synthesis. The vacuum-based deposition methods are limited to fabrication of thin films with highest-level control of crystal quality and composition. Although high power factors can be achieved using these approaches, the main drawback lies in its high cost and low productivity. In comparison, wet-chemical synthesis is more cost-effective for massive production of both thin films and bulk materials. More importantly, wet-chemical synthesis offers easy control over size, shape and composition of the 2D materials and it is also convenient to introduce dopants in the synthetic process to fine-tune and optimize the thermoelectric properties of the prepared 2D materials.

#### Chemical Vapor Deposition

2D layered IV–VI chalcogenides have attracted great attention for the electronic applications due to their band gaps that can facilitate the carrier movement in the 2D network. As a most representative example, SnSe has been widely studied for solar cell and optoelectronic devices [171, 172]. Owing to its layered structure, the *κ*_L_ of SnSe could be reduced significantly to as low as 0.2–0.3 W m^1^ K^−1^ at 800 K [173]. Meantime, SnSe has a semiconducting energy gap of ≈ 0.86 eV and in general a very low *σ* (10^–5^ to 0.1 S cm^−1^). Therefore, CVD has been used to introduce extra elements to enhance the carrier concentration so as to improve its *σ* [174–176]. However, doping certain elements to SnSe via vapor deposition is rarely studied because of its extreme growth process conditions.

**Fig. 12:**
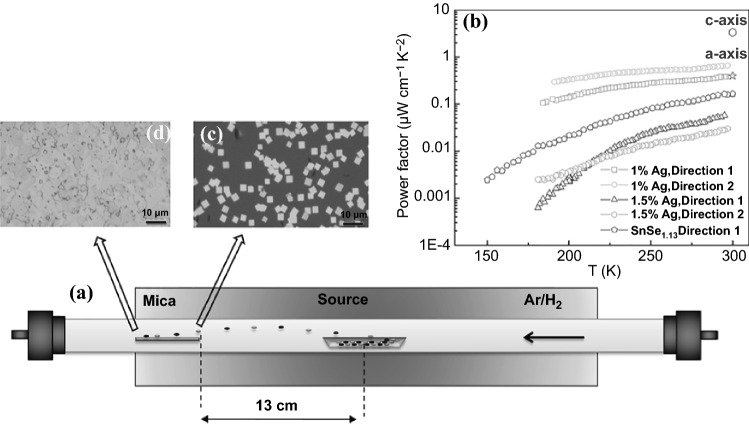
**a** SnSe structure and CVD growth. **b** *PF* of SnSe and Ag doped SnSe nanoflakes. **c** SnSe nanoflakes grown in the high-temperature area on substrate. **d** nanostructured thin films formed in the low temperature area on substrate. Adapted with permission from Ref. [177]

Recently, Gao et al*.* reported a facile CVD approach to grow and dope SnSe nanoflakes, and fabricate the nanostructured thin films (Fig. 12) [177]. The nanostructured structure belongs to the Pnma space group with a layered structure along a-axis, enabling the CVD growth-based nanostructure favorable towards one direction. This is of great interest for TE performance as it potentially moves the carrier towards one particular direction. The CVD growth of SnSe nanoflakes was conducted using 99.999% SnSe powder on the Si wafer with 300 nm thick silicon oxide in the temperature range of 750 to 550 K. Figure 12c, d show the SnSe nanoflakes formed on the surface of the substrate, illustrating that the uniform of nanoflakes were generated during the process.

Upon obtaining the nanoflakes, Gao’s group prepared SnSe_1.13_ and SnSe_0.75_ aiming to study how the ratio difference affects the TE performance [177]. Se deficiency in SnSe_0.75_ contributed to a much higher hole density with a lower hole mobility when compared with SnSe_1.13_, showing poorer TE performance. In the case of SnSe_1.13_, the obtained highest *σ* and the *S* were about 2.5 S cm^−1^ and 300 µV K^−1^, making the *PF* at the level of 0.16 µW cm^−1^ K^−2^ (Fig. 12b), which is about 5% of the *PF* of SnSe single crystals. The low *PF* value could be due to the low carrier mobility and carrier concentration. The same group also doped Ag atoms into SnSe at different ratios and observed that a doping level at about 1% could significantly improve the *σ* and *S*. The highest *σ* and *S* could be up to 4 S cm^−1^ and 370 µV K^−1^ respectively, making the *PF* to be improved to 0.66 µW cm^−1^ K^−2^, four times of the value of undoped sample. Besides, the synthesized SnSe samples could possess a low *κ*, which could further improve the *ZT* value.

#### Molecular Beam Epitaxy

Molecular beam epitaxy (MBE) is widely used to generate 2D layer nanostructures [178–180] on the solid layer of materials, such as silicon [181, 182]. In this area, the preparation of a 2D layer of semiconducting metals or alloys becomes very attractive. Mori et al*.* fabricated Mg_2_Sn (111) thin film on a sapphire c-plane using pure Mg and Sn elements under MBE condition [183]. Below 250 °C, polycrystalline films with an axial preferred orientation were obtained. However, with the increase of the temperature, the loss of Mg element in the thin film made the film towards Sn element rich, which could tune the electronic properties of the Mg_2_Sn thin film. Many other types of semiconducting materials have also been widely studied, including PbTe (111) thin films [184], Bi_2_Se_3_/In_2_Se_3_ superlattices [185–187]. However, the preparation of 2D semiconducting materials using the MBE approach for TE applications is limited. Cecchi et al*.* obtained epitaxial Sb_2+*x*_Te_3_ alloys using MBE to obtain the highest *σ*, *S,* and *PF* at 1810 S cm^−1^, 118 µV K^−1^ and 2.52 mW m^−1^ K^−2^, respectively. Hu et al*.* reported a nanoporous (00l)-oriented Bi_2_Te_3_ nanoplate film made by MBE. In their experiment, pure Bi and Te element were placed in SiO_2_/Si substrates with an oxidized layer of 600 nm. Upon controlling temperature, annealing and hydrothermal process, nanoporous Bi_2_Te_3_ nanoplates was obtained. HRTEM was used to identify the nanopores in the nanoplates. It was observed that the nanoplates film was changed from intersected to tiling, weakening the carrier scattering along the plane and the carrier mobility. The increased carrier mobility thus could increase the *σ*. The carrier concentration of nanoplate films varied with the annealing time also. When the carrier concentration dropped to about 10^20^ cm^−3^, the *S* increased to 187.3 mV K^−1^. This trade-off phenomenon was commonly observed between the *σ* and the *S*. In addition, ethylene glycol was used to treat different Bi_2_Te_3_ nanoplates and it was observed that the boundary density of the pores is the dominant factor to affect the TE performance rather than the total area of the pores because of the boundary density affecting the carrier scattering.

#### Wet Chemistry Method

CVD and MBE could be used to synthesize various semiconducting nanostructure, but some limitations still remain, such as high energy consumption and difficult to control the size of nanostructures. Compared to CVD and MBE, the solution-based chemical synthesis possesses great gains, such as low cost, low energy consumption and easy scaling-up. More importantly, chemical synthesis is able to control size, morphology, composition of the 2D materials more easily by regulating delicate reaction parameters such as temperature, concentration catalyst and dispersants. In this section, the chemical synthesis including hydrothermal, solvothermal and solution chemical synthesis for the TE materials will be summarized in details.

##### Hydrothermal Method

**Fig. 13:**
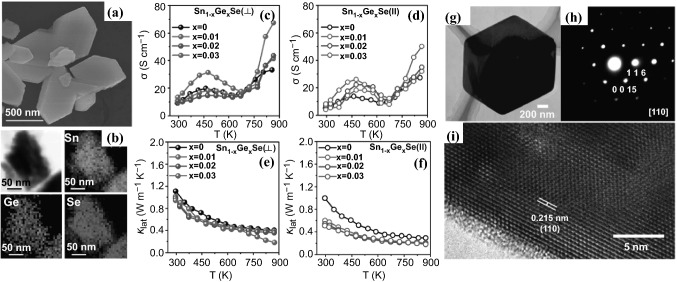
**a** FESEM image of Sn_0.97_Ge_0.03_Se nanoplates. **b** STEM image of Sn_0.97_Ge_0.03_Se nanoplates along with EDAX color mapping for Sn, Ge, and Se. Temperature-dependent **c**, **d** electrical conductivity and **e**, **f** lattice thermal conductivity of Sn_1−*x*_Ge_*x*_Se measured parallel (open symbols) and perpendicular (solid symbols) to the SPS pressing direction, respectively. **g** TEM image, **h** SAED pattern and **i** HRTEM image of a single Sb_2_Te_3_ nanoplate. Adapted with permission from Refs. [192, 193]

Aqueous solution-based hydrothermal synthesis has been widely used for the synthesis of 2D type transition metal dichalcogenides [188–190]. Hydrothermal synthesis of 2D materials can provide different structure and morphology, affording a new pathway to change the carrier mobility and concentration, making the synthesized 2D materials as a good candidate for TE applications. Recently, Chen et al*.* reviewed the hydrothermal method for preparation of various SnSe structures with different dopants for TE studies [191]. Biswas et al*.* reported 2D nanoplates of Ge-doped SnSe synthesized by the hydrothermal approach together with the SPS process [192]. SnCl_2_·2H_2_O and GeI_4_, NaOH and Se powder were placed in a Teflon-lined stainless steel autoclave at 130 °C for 36 h to afford the Sn_1−*x*_Ge_*x*_Se (x = 1–3 mol%) nanoplates (Fig. 13a, b) with a very high yield. Unlike CVD and MBE, this simple synthesis method could be potentially scalable. The as-prepared sample was then densified by the SPS process at 450 °C to afford the sample for TE performance evaluation. The Ge was doped to the SnSe 2D structure to enhance the carrier concentration in the crystalline structure. TEM revealed that the range of lateral dimension of SnSe nanoplates was within 0.5 to 1.0 μm. HRTEM also found that lattice spacing between two apparent planes was estimated to be 3.07 Å, indicating a set of planes (011) favorable in the structure. The TE performance of the synthesized 2D SnSe nanoplates was evaluated from both directions of || (parallel) and ⊥ (perpendicular), and their *σ* and *κ*_L_ are summarized in Fig. 13c–f. Figure 13c, d show that both parallel and perpendicular directions possess the similar *σ* and the trend is also similar starting from semiconducting type to metallic type. The introduction of Ge element enhanced the carrier concentration which significantly improved the *σ*. The *S* was also evaluated for both parallel and perpendicular directions. The *S* increased in positive correlation with temperature, and reached to a maximum value from 550 to 650 K, consistent with solution-processed SnSe samples. A highest *PF* was obtained at ∼5.10 mW cm^−1^ K^−2^ at 3 mol% Ge in SnSe at 873 K. It was observed that synergistic interaction of lattice anharmonicity, point defects, nanoscale grains, and precipitates reduced the *κ*_L_ (Fig. 13e, f) in both parallel and perpendicular directions, which assisted in the enhancement of *ZT* up to ∼2.1 at 873 K.

Zhang et al*.* reported a facile and rapid synthesis method of Sb2Te3 hexagonal nanoplate using hydrothermal treatment in the absence of organic solvent or additive [193]. In the synthesis of this nanoplate, SbCl3 and tartaric acid, NH3⋅H2O solution, K2TeO3, and N2H4⋅H2O were placed in an autoclave, and the reaction temperature was controlled at 180 °C for 5 h, followed by filtration to obtain the Sb2Te3 nanoplates. Figure 13g shows a HRTEM image of a typical single Sb_2_Te_3_ hexagonal nanoplate. As seen in Fig. 13h, the Sb2Te3 nanoplate is a well-crystallized single crystal. Figure 13i displays a well-resolved 2D lattice fringe with a plane spacing of 0.22 nm, in accordance with the lattice planes of (110) in rhombohedral Sb2Te3 nanoplates. The hydrothermal reaction conditions to obtain the Sb_2_Te_3_ hexagonal nanoplate are very critical and it requires to be well-controlled to avoid the formation of nanoparticle or NW. Using the home-made setup, the *S* of Sb_2_Te_3_ nanoplate was evaluated to be 125 mV K^−1^ as p-type semiconductor using a home-made setup, which is higher than these of other type nanocrystals, such as Sb_2_Te_3_ nanoparticles and nanorods. Due to the anti-site effect derived from Sb atom on the Te lattice sites, the *S* is approximately 1.6 times of that value of undoped bulk crystals at 79 mV K^−1^, which is attributed to the energy filtering due to the nano-boundaries created by the extra atom throughout the doping process.

##### Solvothermal Method

As one of the best TE materials, antimony telluride (Sb2Te3) has been well investigated as a p-type semiconductor [30, 194]. However, the study of how the shape and size changes in 2D type Sb2Te3 nanomaterials is limited. Lee et al*.* reported a solvothermal approach to prepare the Sb2Te3 nanoplates. SbCl3, K2TeO3 and PVP were dissolved in diethylene glycol, followed by the addition NaOH aqueous solution. Upon the solvothermal process in a Teflon-lined stainless-steel autoclave at 230 °C for 24 h, the corresponding nanoplates were obtained. SEM analysis showed the edge lengths and thicknesses of the nanoplates are 3–5 μm and ∼100 nm respectively. Sequentially, the TE performance was evaluated in comparison with the sample prepared by the ball milling. Interestingly, the *κ* of the Sb2Te3 nanoplates is much lower than that of samples obtained from the ball milling process. Such a low *κ* could be attributed to the grain boundary generated between nanoplates. The *S* is in the range of 300–350 μV K^−1^, about two times higher than those of Sb2Te3 prepared by the melting method (102–144 μV K^−1^). The improvement in the *S* can arise from the energy filtering effects caused by the boundaries between Sb2Te3 nanoplates.

**Fig. 14:**
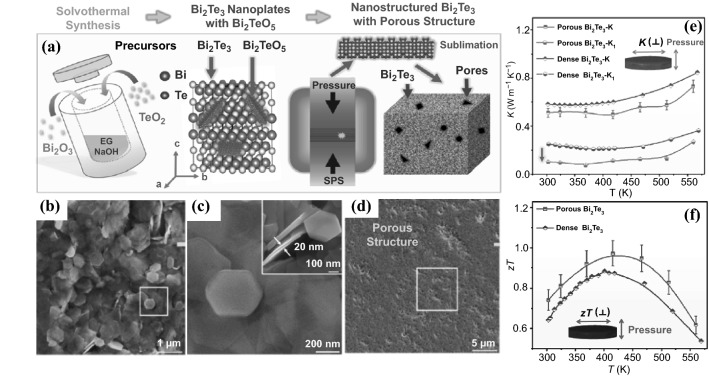
**a** Illustration of the solvothermal synthesis of Bi_2_Te_3_ nanoplates followed by SPS to fabricate a porous nanostructured Bi_2_Te_3_ pellet. SEM images of **b, c** the as-synthesized Bi_2_Te_3_ nanoplates and **d** the as-sintered nanostructured porous Bi_2_Te_3_. Temperature dependence of **e** *κ* and *κ*_L_, **f** ZT of the as-sintered nanostructured porous Bi_2_Te_3_ pellet compared with dense nanostructured Bi_2_Te_3_. Adapted with permission from Ref. [197]

As another important class of semiconducting materials, 2D Bi_2_Te_3_ nanostructures have attracted great attention to enhance the TE performance. Takashiri et al*.* used Bi_2_O_3_ and TeO_2_ under basic conditions via a solvothermal process to obtain the Bi_2_Te_3_ nanoplate, followed by electrical deposition and annealing to afford the electrodeposited layers [195, 196]. Annealing at 250 °C helped reduce the boundaries between nanoplates, and further enhanced the *σ* and *S* by about 200% and 50%, respectively. Thermal annealing improved the crystallinity of electrodeposited layers, which decreased the number of defects (carrier concentrations) and the number of boundaries (increasing mobility). The structure variation arising from the post treatment could significantly improve the TE performance through tuning the carrier concentration and mobility. Chen et al*.* reported a solvothermal method to introduce high porosity in Bi_2_Te_3_ hexagonal plates and decrease the overall *κ* as well as the *κ*_L_ [197]. Through reduction of the *κ*, the TE performance could be enhanced significantly when the *PF* remained unchanged. In the synthesis of Bi_2_Te_3_ nanoplates (Fig. 14a**)**, Bi_2_O_3_ and TeO_2_ were placed in an autoclave in the presence of NaOH and ethylene glycol at 210 °C for 24 h. The Bi_2_Te_3_ nanoplates were obtained through filtration. Then the obtained Bi_2_Te_3_ nanoplates were subjected to the SPS process and sublimation to obtain the sample for the structure characterization. SEM analysis showed the Bi_2_Te_3_ nanoplate has the thickness of 20 nm (Fig. 14b, c) and can induce a high density of grain boundaries in the pellet after the sintering. As shown in Fig. 14d, the sintering process could introduce the density of pores in the structure and the pore size is at a size of ∼400 nm in the matrix. Sequentially, the *σ* and *S* were measured in comparison with the dense sample. It was found that the similar value was obtained between the dense sample in other literature and the as-prepared porous structure, leading to a similar magnitude of the *PF*. The porous structure in the matrix could significantly reduce the *κ*_L_ to less than 0.1 W m^−1^ K^−1^ (Fig. 14e) due to the phonon gas theory. Owing to the overall reduction in the *κ*, the *ZT* value was improved dramatically up to 0.97 at 420 K (Fig. 14f), the highest values reported for pure n-type Bi_2_Te_3_ semiconductors.

Zou et al*.* reported a microwave-assisted solvothermal synthesis method to obtain Bi_2_Te_3−*x*_Se_*x*_ nanoplates which could enhance the TE performance [198]. In the synthesis of Bi_2_Te_3−*x*_Se_*x*_ nanoplates, Bi(NO_3_)_3_·5H_2_O, Na_2_TeO_3_ and Na_2_SeO_3_ were placed in reaction vessel in the presence of ethylene glycol and NaOH at 230 °C for 5 min. During the synthesis, various loadings of Se were doped in the samples to substitute the Te element to study its impact on the TE performance. The obtained Bi_2_Te_3−*x*_Se_*x*_ nanoplates were subjected to the SPS process to make the pellets for the TE measurement. It was found that the loading of Se element doesn’t seem to have huge impact on the overall performance, leading to the *PF* in the range of 1.5–1.9 mW m^−1^ K^−2^. The texture fraction in the nanostructure materials is smaller than that of the bulk polycrystalline counterpart, and can help reduce the phonon scattering, leading to the reduction in the *κ*. A lowest *κ* of 0.69 W m^−1^ K^−1^ value was achieved in the Bi_2_Te_2.7_Se_0.3_ pellet, which is much lower than the value between 1.5 and 2 W m^−1^ K^−1^ of their bulk counterpart materials. Such reduction is attributed to the nanostructure grains and boundaries in the pellet made by the Bi_2_Te_3−*x*_Se_*x*_ nanoplates. There are mainly two factors promoting the reduction of the *κ* namely the Umklapp phonon–phonon scattering by the inherently strong anharmonicity and the wide frequency phonon scatterings caused by the multi-scattering pathways. The same group also performed the theoretical studies on the thermal transport and phonon transport to understand the rationale behind the *κ* reduction, revealing that the complex carrier scatterings helped suppress the bipolar effect and weakened the dependence of transport properties on the carrier movement.

##### Solution-Phase Synthesis

Hydrothermal and solvothermal approaches have been used to grow 2D type inorganic semiconducting materials. These two methods usually require the reaction to be performed at high temperature (e.g., from 150 to 300 °C) in a sealed stainless vessel. At this reaction condition, the inorganic species in general can grow in a specific direction to form the 2D type of materials. As another approach, solution chemical synthesis can be used to prepare 2D semiconducting materials with a lot of benefits, including tunable reaction conditions and easy scale-up. Cabot and co-workers reported a chemical synthesis method using tin selenide molecular precursor under solution process to prepare 2D SnSe nanoplate for TE studies [199]. In the synthesis of 2D SnSe nanoplate, tin chloride selenium dioxide, tri-n-octylphosphine and oleyamine were premixed in a very short of time, followed by the addition of oleic acid to produce dentritic SnSe nanostructures. Then the SnSe precursor was injected into a reaction flask preheated at 420 °C for decomposition to prepare the SnSe nanostructures. TEM and SEM characterization of SnSe nanostructure showed that as-prepared nanoplates had the size of 4 ± 1 μm and a thickness of 90 ± 20 nm and the crystal followed the [100] crystal direction oriented along the pressure axis by XRD analysis. TE property study revealed that both cross-plane and in-plane afforded very similar *σ* of 15–30 S cm^−1^. A slightly higher *S* of 400 µV K^−1^ was obtained in the cross-plane direction, which could be attributed to the energy filtering introduced by the grain boundaries, specifically the preferential scattering of the low-energy carriers at the plate interfaces. A very low *κ* was obtained in both directions, significantly improving the *ZT* value of the SnSe nanoplates. The low *κ* could be arisen from the low *κ*_L_ because of the phonon scattering.

Biswas et al*.* reported a chemical synthesis to prepare several n‑type ultrathin layers of Bi doped SnSe nanosheets [200, 201]. In the synthesis of SnSe nanosheets, SnCl_4_·5H_2_O and SeO_2_ were taken in a mixture of oleylamine and 1,10-phenanthroline. The reaction was performed at 200 °C under N_2_ followed by filtration to afford the nanostructured materials at the gram scale. Bi precursor was used in the reaction to dope the nanosheet to afford Sn_0.94_Bi_0.06_Se. Powder X-ray diffraction analysis shows the 2D SbSe nanosheet has the lattice parameters of a = 11.50 Å, b = 4.15 Å, and c = 4.45 Å and the indirect band gap was measured to be ∼0.88 eV. Field emission scanning electron microscope (FESEM) showed that the Bi doped SnSe nanosheets possessed a thickness of 1.2–3 nm. Such a dimension is critical to create the nanoscale grain boundaries and point defects across the interphase between Se-Se layers. Such boundaries are effective for the phonon scattering and improvement of TE performance. Based on the Hall Effect measurement, the introduction of Bi element in the SnSe could significantly enhance the carrier concentration, leading the *σ* up to 12.5 S cm^−1^ at 750 °C for the cross-plane direction. Negative *S* was observed for both cross- and in-plane directions. The highest *PF* of 100 μW m^−1^ K^−2^ was obtained for Sn_0.94_Bi_0.06_Se nanosheets, much greater than that of SnSe. More interestingly, the nanoboundaries in the nanosheet could facilitate the phonon scattering, leading to a low *κ*_L_ of ∼0.3 W m^−1^ K^−1^. This low *κ* could contribute significantly to the *ZT* enhancement to 0.21 at about 700 °C for Bi doped SnSe nanosheets. Similarly, Hu et al*.* reported a chemical synthesis method using SnCl_2_⋅2H_2_O and Se in the basic solution to prepare SnSe 2D nanosheets [202]. In their work, extremely low *κ* of 0.09 W m^−1^ K^−1^ was obtained, demonstrating the thin film made from 2D nanosheet could be used for low-grade waste heat recovery.

Zou et al*.* reported a chemical method to dope Te element into the SnSe nanosheets to enhance the TE performance [203]. In the synthesis of SnSe nanosheets, SnCl_2_⋅2H_2_O, Na_2_SeO_3_, and Na_2_TeO_3_ were added into the NaOH solution in ethylene glycol. After the stirring, the reaction was heated at 230 °C for 10 min to obtain the Te doped SeSe nanoplates. SEM and TEM analysis shows the nanoplates have the average lateral size and thickness of about 10 µm and 200 nm, respectively. With the increasing Te element in the SnSe nanoplates, the *S* dropped from 365 to about 341 µV K^−1^. Due to the trade-off relationship between the *σ* and *S*. The *σ* improved doubly from 50 to about 100 S cm^−1^. The Te element in the alloy could reduce the band gap and the hole concentration, resulting in a stronger bipolar effect, and thus increase in the *σ* and reduction in the *S*.

Another noteworthy point is that with increasing Te element into the alloy, the point defects were introduced, which enhanced the phonon scattering and led the *κ* to be reduced by about 30% overall. In this case, the average *ZT* of 0.58 was obtained. Gregory et al*.* reported a similar method to synthesize SnSe nanoplates using water as solvent without using any surfactant [204]. The *PF* is eightfold higher than that of material made using citric acid as a structure-directing agent. As another type of important semiconducting materials, 2D Bi_2_Te_3_ nanostructure is also well-studied, especially in 1D type. Hyeon et al*.* reported a chemical method to prepare n-type of ultrathin Bi_2_Te_3_ nanoplates with the improved TE performance [205]. In the synthesis, bismuth neodecanoate and tri-n-octylphosphine-tellurium were used as Bi and Te sources in polyamine, respectively. 1-Dodecanethiol was used with the proper concentration to form the Bi_2_Te_3_ nanoplates. The thickness of the nanoplates was about 1 nm. Sequentially the as-prepared nanoplates were sintered into pellets for the TE performance measurement. The *S* was in the range of − 130 to − 160 μV K^−1^. It was noteworthy that the electron concentrations and mobility varied at different sintering temperatures possibly because of the interphase effect, which facilitated the carrier scattering and defects by the organic residue from the synthesis. Meantime, the *κ* was observed to be reduced significantly (lowest at about 0.4 W m^−1^ K^−1^). This phenomenon was also observed in other 2D materials and this can be attributed to the scattering of carriers and boundaries between the 2D materials.

### 3D Nanostructure

One simple approach to improve TE property in bulk materials is to induce the formation of porosity. Although the traditional approach to TE materials was to create bulk samples with very little remaining porosity (> 99% relative density), many recent studies have shown that either random or template-assisted uniform porosity can be beneficial for TE performance when properly optimized [21, 206]. Reported studies have shown that the sizes of the pores in TE materials and their distribution pattern play critical role in TE properties. To maintain sufficient *σ*, these structures generally must be sintered to at least 80% theoretical density, effectively limiting the porosity.

#### Random Porous Structure

Randomly porous structures are created by deliberately partially densifying a material to generate a distribution of pores throughout the structure. Randomly porous structures are easily synthesized but the effects of nano-structuring on the *κ* (and in some cases, the *S*) are much weaker due to the lack of morphological control.

**Fig. 15:**
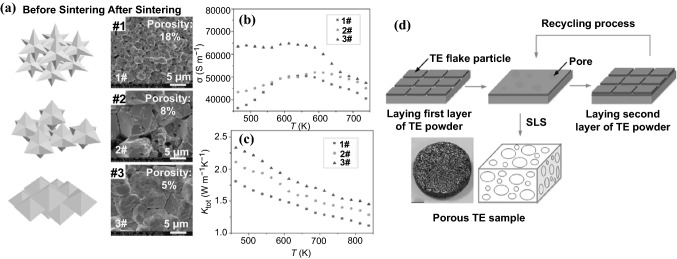
**a** Schematic illustration (left) and SEM images (right) of the as-sintered porous monoliths from 1# (hexapods), 2# (less-protruding hexapods) and 3# (octahedra) PbS nanocrystals. Temperature dependence of **b** *σ* and **c** κ of the three sintered TE monoliths. **d** Schematic diagram of selective laser sintering 3D printing process to prepare porous TE monoliths. Adapted with permission from Refs. [207, 208]

In 2018, Xu et al*.* synthesized PbS nanocrystals with different shapes and consolidated them into highly porous and well crystalline monoliths using the SPS process (Fig. 15a) [207]. It was found the relative density and TE performance of the porous PbS monoliths could be tuned simultaneously (Fig. 15b, c). The as-obtained porous monolith with a large grain size has low relative mass density (82%) and high porosity, and also exhibits a high *σ*, a low *κ*, and hence an excellent *ZT* (1.06 at 838 K). The origin of high *ZT* was studied by DFT calculations, indicating that high *ZT* can be attributed to enhanced scattering of phonons caused by the porous structures.

In 2019, Shi et al*.* employed a 3D printing technique (Fig. 15d) to fabricate porous bismuth antimony telluride (Bi_0.5_Sb_1.5_Te_3_, BST) and its TE properties were studied [208]. The porous TE samples were fabricated with the selective laser sintering method using 100 mesh BST powder. Energy density of the laser light that scans across the thin layer of TE powder was carefully controlled to ensure that TE powder was only partially melted to form the desired porous TE material. The laser sintering process of BST particles led to the formation of many micrometer- and nanometer-sized random pores, accounting for the reduction of the *κ*. The minimum *κ* of the porous BST samples was measured to be 0.27 W m^−1^ K^−1^ at 54 ℃. The reduction in the *κ* is attributed to the boundaries and defects formed during the selective laser sintering process as well as the porous structures of the materials. The *ZT* of the porous samples was found to have a maximum value of about 1.29 at 327 K, higher than that of the BST bulk material.

#### Template-Assisted Uniform Porous Structure

**Fig. 16:**
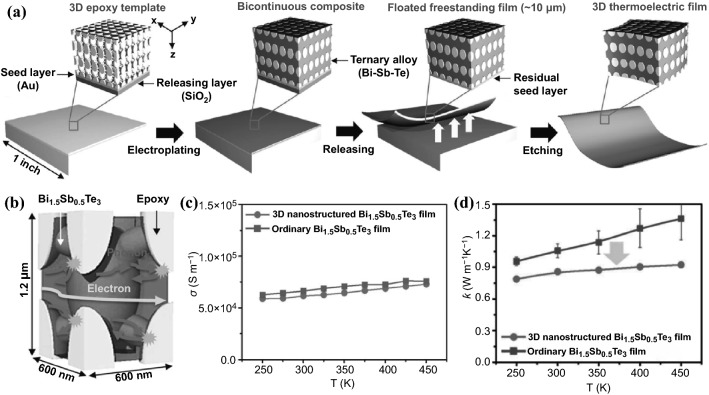
**a** Schematic illustration of procedures for the fabrication of a freestanding and transferrable 3D nanostructured TE film. **b** Structural unit cell of a 3D nanostructured Bi_1.5_Sb_0.5_Te_3_ film illustrating the concept of increasing phonon boundary scattering without altering electrical transport. Comparison of **c** electrical conductivity and **d** thermal conductivity of the 3D nanostructured and ordinaryBi_1.5_Sb_0.5_Te_3_ films as a function of temperature. Adapted with permission from Ref. [209]

In 2017, Hong et al*.* reported the preparation of a periodic 3D nanostructured TE monolith, with an approximate pore size of 140 nm, by electrochemical deposition of a Bi–Sb–Te ternary alloy into a highly ordered, interstitial porous network in an epoxy template predefined by advanced lithography (Fig. 16a) [209]. The electroplating conditions for the 3D nanoconfined geometry was optimized to facilitated the uniform and dense filling of Bi_1.5_Sb_0.5_Te_3_ into the template over a large area of 625 mm^2^. Most nanostructured TE materials suffer from undesirable degradation of the σ. In contrast, the 3D nanostructures, however, are able to maintain electron transport properties because the sizes of the TE struts in a structural unit cell are sufficiently larger (140 nm) than the mean free path of electrons (~ 40 to 60 nm). It is suggested that the extrinsic phonon scattering at the interfaces of the nanostructures without changing electrical transport is responsible for the selective reduction of the *κ* while the *S* and *σ* are almost intact (Fig. 16b). The 3D nanostructure successfully resulted in a decreased *κ* from 1.14 to 0.89 W m^−1^ K^−1^, at 350 K while maintaining a *σ* of 644.5 S cm^−1^ and a *κ* of 144 mV K^−1^ (Fig. 16c, d). The 3D nanostructured Bi_1.5_Sb_0.5_Te_3_ film showed an improved *ZT* value of 0.56 at 400 K, which was approximately 50% higher than the value for an ordinary Bi_1.5_Sb_0.5_Te_3_ film. In 2018, the same group also reported the deposition of nanoscaled ZnO film on the 3D nanostructured epoxy template via the atomic layer deposition [210]. In this work, the suppressed *κ* of the 3D ZnO film is ∼3.6 W m^−1^ K^−1^ at 333 K, which is ∼38 times lower than that of the blanket ZnO film.

### Nanoscale Doping in Bottom-up Process

**Fig. 17:**
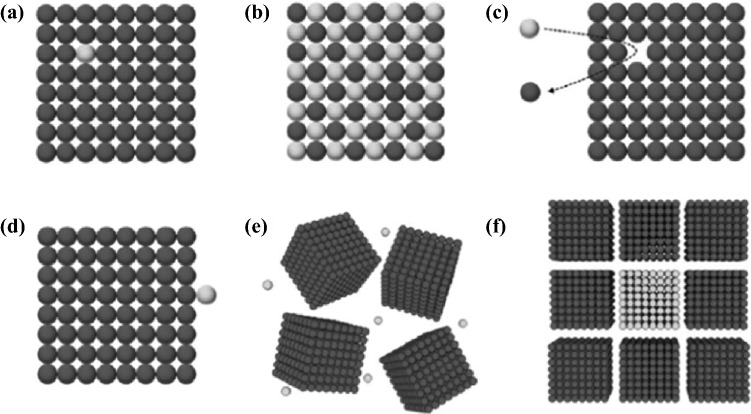
Scheme of possible strategies to tune doping in nanoparticle-based solution-processed nanomaterials: **a** extrinsic dopants during synthesis; **b** stoichiometry control; **c** post-synthesis atomic substitution; **d** surface chemistry; **e** dopant additives during consolidation; **f** modulation doping. Adapted with permission from Ref. [22]

For semiconductor thermoelectric materials, the introduction of small quantities of impurity (element doping) during the bottom-up preparation can not only tailor the carrier concentration and/or mobility to optimize the σ, but also induce point defects (vacancies or self-interstitials) and adjust the microstructures (e.g., phase separation, formation of nanoscaled precipitates or ultra-fine grains) to reduce the *κ*_L_ [211–220]. Although the carrier concentration in a material can be modulated using defect-engineering, or stoichiometry control during synthesis, reaching the optimized carrier concentration (typically 10^19^ cm^−3^) often require additional extrinsic doping. Intuitively, this 10^19^ carrier concentration is equivalent to about 1–30 electronically active sites in a 10 nm particle. Schematic illustrating doping mechanisms in nanostructures is shown in Fig. 17. The most commonly used strategy in modulating the carrier concentration at the nanoparticle level is the incorporation of dopants at the initial reaction solution [221]. In this approach, the dopants and host precursors are homogeneously mixed before the nucleation, resulting in uniform doping. However, not all dopants can be incorporated using this method. The coordinating ligands and proper control of reaction conditions are critical to enable high doping efficiency of these extrinsic elements rather than just a secondary phase or nanocomposite. In addition, surfactants, which can be used to modulate surface energies, can either inhibit or promote the incorporation of external dopant species. Generally, the addition of any type of impurities will affect the growth kinetics and thermodynamics of these nanostructures. Therefore, understanding the solubility and kinetics of the dopants and the host is very important to control the optimal amount of dopant that can be incorporated into these materials.

Alternatively, dopant ions can be introduced within the preformed nanoparticles. Ion exchange processes can be employed in this case, which is akin to ion-implantation in semiconductor processing. Although this is a more expensive strategy, a non-equilibrium thermodynamic doping process can be achieved, thus making it possible to introduce a large amount of dopant beyond the solubility limit allowed by thermodynamics [222]. Besides introducing impurities, a common strategy to tune carrier concentration in nanoscale level (especially in 2D structures) is by applying gate-voltage [223]. In essence, gate voltage not only provides carrier concentration modulation, but can also be used to alter the carrier scattering mechanism, resulting in multi-fold enhancements of thermoelectric properties by enhancing electrical conductivity and reducing thermal conductivity.

## Conclusion and Outlook

In this review paper, we provided a comprehensive review on the state-of-the-art strategies and bottom-up approaches that had been employed for constructing nanostructured semiconductor TE materials with different dimensions, focusing on the relationships between the structures and the key electronic and thermal transport parameters contributing to *ZT*, such as the *S* enhancement due to the energy filtering or quantum confinement effects, and the *κ* reduction inherently due to the phonon scattering. Table 1 summarizes the TE properties of various nanostructured materials prepared via bottom-up approaches. The vapor-based processes are expected to play an important role in preparing high-precision materials for theoretical mechanism studies and/or fabrication of micron-scale TE devices, while the solution-based preparation methods with controllable size, morphology and surface chemistry offer a convenient route towards inexpensive and scalable low-dimensional TE materials. However, the practical adoption of those liquid-based processes towards high quality nanomaterials still remains a challenge. Scale-up production of these nanostructured materials inevitably requires automated processes with nearly 100% yields, high nanoparticle concentrations in solution, low cost and environmentally-benign precursors, solvents, reductants and surfactants, as well as the recycling of solvents and possible side-products. Moreover, the consolidated conditions which may cause significant atomic and interface redistribution should also be investigated and optimized to assemble the nanostructured materials with a maximum retention of their electronic and phononic characteristics. Deep fundamental understanding of the electronic and thermal transport properties of these nanostructured materials will be highly valuable for further development of advanced, practical and high-performance TE devices. In addition, through developments in low-dimensional materials, some promising concepts such as nano-porous structures have been applied and have seen some early successes in traditional 3D bulk materials. Remarkably, it has been shown that it is in fact possible for porous structures to be 3D-printed through selective laser sintering, which results in an enhanced *ZT*. Moving forward, to realize widespread commercial interest, several key factors need to be taken into account in general: low-cost and scalable processing methods, earth-abundant and non-toxic materials to ensure sustainability, easy device integration (i.e. ensuring adequate performance on the device level). Therefore, any breakthrough in any of the aforementioned aspects will undoubtedly be an important step forward towards making TE devices more competitive.

**Table 1:** Summary of properties of TE materials prepared via various bottom-up methods

Process type	TE Materials	Mechanism on property improvement	*σ* (S cm^−1^)	*S* (μV K^−1^)	*κ* (Wm^−1^ K^−1^)	*PF* (μW m^−1^ K^−2^)	ZT_max_	Temp. (K)	Refs.
0D nanoparticles & nanoinclusion									
Colloidal synthesis	PbSe quantum dot films	Quantum confinement, phonon scattering	~ 240	~ 425	~ 1.15	–	1.37	400	[83]
	PbTe/PbS nanocrystal films	Quantum confinement, phonon scattering	~ 21	~ 490	~ 0.675	~ 450	0.3	405	[84]
	PbTe@PbS core–shell nanoparticle pellets	Phonon scattering	125	− 185	0.53	–	1.03	710	[86]
Hydrothermal synthesis	SnTe nanocrystal pellets	Phonon scattering energy filtering	600	90	0.60	350	0.49	803	[91]
	Cu_2_S microcrystal pellets	Phonon scattering	100	120	0.2	–	0.38	573	[92]
Electrodeposition process	Bi_2_Te_3_ nanocrystals on PEDOT films	Phonon scattering	691	− 146	–	1473	–	298	[96]
	Gold nano-particles-BiTe composite pellets	Phonon scattering	71	− 380	0.5	–	0.62	298	[98]
Nanoinclusion	Pt nanoparticles in Bi_2_Te_2.7_Se_0.3_thin films	Energy filtering, phonon scattering	720	220	–	3510	–	298	[107]
	Ag nanoparticles in Bi_2_Te_3_ nanopowder pellets	Energy filtering, phonon scattering	525	− 140	0.58	–	0.77	475	[108]
	Ag nanocrystals in PbS nanocrystal pellets	Energy filtering, phonon scattering	660	− 200	0.8	> 1000	1.7	850	[109]
1D Nanowires/nanofibers/nanotubes									
Solution-phase synthesis	Bi_2_Te_3_ NW pellets	Phonon scattering	430	− 240	1.0	2520	0.96	380	[116]
	PbTe NW pellets	Phonon scattering	95	295	0.9	850	0.33	350	[117]
	Bi_2_Te_2.7_Se_0.3_ NW pellets	Phonon scattering	465	− 160	0.46	1023	0.75	320	[122]
	Te–Bi_2_Te_3_ NW pellets	Energy filtering, phonon scattering	5.244	588	0.309	~ 300	0.236	400	[120]
	PbTe–Bi_2_Te_3_ NW pellets	Energy filtering, phonon scattering	100	− 310	0.51	~ 950	1.2	620	[124]
	Ag_2_Te–Bi_2_Te_3_ NW pellets	Energy filtering, phonon scattering	28	275	0.2	210	0.41	400	[123]
Template-assisted electrodeposition method	Bi_0.5_Sb_1.5_Te_3_ NW membranes	Phonon scattering	78	138	–	153	–	298	[143]
	Bi_38_Te_55_Se_7_ NW thin films	Phonon scattering	2200	− 115	–	2820	–	298	[149]
	Bi_15_Sb_29_Te_56_ NW thin films	Phonon scattering	720	156	–	1750	–	298	[149]
Electrospinning	PbTe nanotube mats	Quantum confinement, phonon scattering	0.148	196	–	0.567	–	298	[163]
2D Nanoflake/nanosheet /nanoplate									
Chemical vapor deposition	Ag-doped SnSe nanoflake thin films	Phonon scattering	5	370	–	66	–	300	[177]
Molecular beam epitaxy	Sb_2.31_Te_3_ thin films	Phonon scattering	1810	118	–	2520	–	298	[224]
Hydrothermal method	Ge-doped SnSe nanoplate pellets	Phonon scattering	70	275	0.18	510	2.1	873	[192]
Solvothermal method	Bi_2_Te_3_ nanoplate films	Phonon scattering	122	− 103	–	128	–	298	[196]
	Bi_2_Te_3_ nanoplate pellets	Phonon scattering	600	− 137	0.1	1057	0.97	420	[197]
	Bi_2_Te_3_ nanosheet pellets	Phonon scattering	735	− 180	1.2	2400	0.69	333	[225]
	Bi_2_Te_2.7_Se_0.3_ nanoplate pellets	Phonon scattering	480	− 198	0.72	1875	1.23	480	[198]
Solution-phase synthesis	SnSe nanoplate pellets	Phonon scattering	31	320	0.55	375	1.05	805	[226]
	Sn_0.94_Bi_0.06_Se nanosheet pellets	Phonon scattering	12.5	− 285	0.3	100	0.21	719	[201]
	SnSe nanoplate pellet	Phonon scattering	35	340	–	400	–	550	[204]
3D porous nanostructure									
Random porous structure	PbS nanocrystal monolith	Phonon scattering	~ 400	~ 187	0.56	1375	1.06	838	[207]
	Bi_0.5_Sb_1.5_Te_3_ powder monolith	Phonon scattering	403	173	0.27	1100	1.29	327	[208]
	Nanoporous SnSe pellets	Phonon scattering	~ 40	~ 325	0.24	506	1.7	823	[217]
Template-assisted uniform porous structure	Bi_1.5_Sb_0.5_Te_3_ film	Phonon scattering	644.5	144	0.89	1250	0.56	400	[209]
	ZnO film	Phonon scattering	~ 45	~ 253	~ 3.6	290	0.072	693	[210]
Nanoscale doping									
Hydrothermal method	Pb, Cd-doped poly-crystalline SnSe	Phonon scattering, carrier concentration	~ 85	~ 285	0.23	750	1.9	873	[215]
Hydrothermal method	Pb, S-doped poly-crystalline SnSe	Phonon scattering, carrier concentration	~ 38	~ 325	0.13	418	1.85	873	[218]
Hydrothermal method	Se quantum dot/Sn _0.99_Pb_0.01_Se nanocomposite	Phonon scattering, energy filtering	~ 31	~ 420	0.245	560	2.0	873	[214]
Solvothermal method	Cd-doped poly-crystalline SnSe	Phonon scattering, carrier concentration	79	295	0.33	690	1.7	823	[227]
Solvothermal method	Cu-doped poly-crystalline SnSe	Phonon scattering, carrier mobility	56	316	0.32	557	1.41	823	[218]
